# Systematic revision of *Paralongidorus* (Nematoda: Longidoridae) based on molecular and morphological evidence, with the description of a new species from Spain

**DOI:** 10.1186/s40851-026-00259-6

**Published:** 2026-02-07

**Authors:** Ana García-Velázquez, Rosana Salazar-García, Carolina Cantalapiedra-Navarrete, Inmaculada Criado-Navarro, Juan E. Palomares-Rius, Pablo Castillo, Antonio Archidona-Yuste

**Affiliations:** Institute for Sustainable Agriculture (IAS), Spanish National Research Council (CSIC), Department of Crop Protection, Avenida Menéndez Pidal s/n, Córdoba, 14004 Spain

**Keywords:** Cytochrome oxidase c subunit 1, D2-D3 of 28S rRNA, Forest, Integrative taxonomy, ITS rRNA, Morphology

## Abstract

The needle nematode genus *Paralongidorus* Siddiqi, Hooper & Khan, 1963 currently comprises 76 species of polyphagous plant ectoparasites with global distribution. Here, we revise the taxonomic ambiguities within the genus and update the polytomous key to facilitate species identification. Integrating ribosomal (D2–D3 expansion segments of 28S, ITS, partial 18S) and mitochondrial (partial *COI* mtDNA) markers, we reassess phylogenetic relationships within *Paralongidorus* and *Longidorus*. Our analyses reveal that *Paralongidorus* is paraphyletic, consistently resolving into two distinct subclades. We describe *Paralongidorus cantabronavarrus* sp. nov. from northern Spain, a species characterized by both molecular placement and morphological traits, including a uniquely anterior vulval position (V = 26–29%), the most extreme reported within the genus. This feature may represent a derived developmental adaptation, with potential ecological significance. Amphidial fovea morphology also shows evidence of convergent evolution, complicating generic boundaries. These findings challenge the validity of *Longidoroides*, reinforce the separation between *Paralongidorus* and *Longidorus*, and highlight the value of integrative approaches in resolving long-standing taxonomic uncertainties. The phylogenetic placement of *P. cantabronavarrus* sp. nov. is consistent across markers and aligns with previous studies. Overall, this work expands our understanding of *Paralongidorus* biodiversity in the Iberian Peninsula and underscores the need for further intensive nematological surveys in this area.

**Supplementary information:**

The online version contains supplementary material available at 10.1186/s40851-026-00259-6.

## Introduction

The needle nematode genus *Paralongidorus* Siddiqi et al. 1963 [1], comprises ectoparasitic species that spend their entire life cycle attached to host plant roots. These nematodes are of scientific and economic interest due to their direct damage to root systems and the role of certain species as vectors of economically significant pathogenic plant viruses [2]. *Paralongidorus* (Nematoda: Longidoridae) is characterised by a long body, a prominent odontostyle, an odontophore with a slight swelling of basal musculature, a distinctive amphidial fovea ranging from funnel- to stirrup-shape, with openings extending approximately as wide as lip region width [3]. Body length varies from 1.7 mm in *P. duncani* Siddiqi et al. 1993 [3] to 14.1 mm in *P. plesioepimikis* Palomares-Rius et al. 2013 [4]. Odontostyle length ranges from 40 µm in *P. duncani* to 227 µm in *P. plesioepimikis* [3, 4]. The genus currently comprises 75 nominally valid species distributed globally [5].

Over recent decades, the taxonomic placement of certain *Paralongidorus* species has been a subject of ongoing debate, primarily due to difficulties in discerning morphology, particularly regarding the amphidial fovea opening, the trait previously considered pivotal for delimiting genera within Longidoridae Thorne, 1935 [6, 7]. The genera *Longidoroides* and *Siddiqia* were established by Khan, Chawla and Saha [8] to accommodate species exhibiting distinct amphidial features: *Longidoroides* was defined by a pouch-like amphidial fovea with a small to large slit-like aperture, whereas *Siddiqia* was characterised by a lip region clearly set off from the body by a deep constriction and the presence of an amphidial fovea typical of *Paralongidorus*. Subsequently, *Brevinema* Stegarescu, 1980 [9], was proposed as intermediate between *Xiphidorus* and *Paralongidorus*, and distinguished by a posteriorly located single guiding ring, an unforked odontostyle base, and a swollen but unsclerotised odontophore base, as observed in *Longidorus pisi* Edward, Misra and Singh, 1964a [10] and *Xiphinema sandellum* Heyns, 1966 [11]. Later, Khan [12] introduced the genus *Inagreius* to encompass species exhibiting morphological traits of both *Siddiqia* (e.g. an expanded lip region, sharply set off by a constriction and a secondary depression posterior to the groove, with a transverse slit-like amphidial aperture) and *Longidoroides* (e.g. a bilobed, pouch-shaped fovea). However, Luc and Doucet [13] considered the establishment of these genera unjustified, arguing that the characters used were minor and that such variation could occur within a single genus, for example, *Xiphinema*. Consequently, they proposed synonymising *Siddiqia* with *Paralongidorus* and *Inagreius* with *Longidoroides*. Coomans [14] accepted these synonymies and also rejected the validity of *Brevinema*, considering it a synonym of *Longidorus*. Subsequently, Siddiqi et al. [3] synonymised *Longidoroides* with *Paralongidorus* based on the amphidial structure of *P. sali* Siddiqi et al. 1963 [1]. Hunt [15] further suggested that all species possessing a transverse slit-like amphidial opening should be classified under *Paralongidorus*. Nevertheless, Coomans [16], upon examining type specimens, found this interpretation unconvincing and rejected the proposed synonymization. This debate was largely confined to a narrow morphological framework; however, it is ultimately essential to adopt an integrative approach, combining modern morphological techniques with genetic and phylogenetic data, to elucidate the evolutionary history and taxonomic placement of the taxa [17].

Despite substantial progress in molecular techniques, morphological and morphometric identification of *Paralongidorus* spp. remain essential for accurate species delimitation. The polytomous key developed by Escuer and Arias [18] continues to serve as a valuable resource for morphological identification; however, after nearly three decades, it warrants comprehensive revision. Nine additional species have been described in the intervening years, and several have been reassigned to *Longidorus* following detailed morphological and scanning electron microscopy (SEM) studies by Decraemer and Coomans [6] and Clavero-Camacho et al. [19]. Despite extensive surveys across diverse biogeographical regions, the taxonomy of *Paralongidorus* remains incompletely resolved. The existence of cryptic species in other longidorids suggests that the numerous global records of *P. maximus* may warrant revision under contemporary taxonomic frameworks. Recent developments in integrative taxonomy, combining morphological diagnostics with molecular phylogenetics, have enabled more robust species delineation within Longidoridae [4, 20–24]. Within this framework, the detection of two previously undocumented *Paralongidorus* populations in forest areas of northern Spain prompted a comprehensive investigation to determine their taxonomic status.

The specific objectives of this study were as follows: (i) to update the list of valid nominal species within the genus *Paralongidorus*, and to revise the polytomous key of Escuer and Arias [18] by incorporating newly described species and excluding those reassigned to *Longidorus*; (ii) to undertake detailed morphological and morphometric characterisation of the two *Paralongidorus* populations collected from forested areas in northern Spain, and to compare them with other recognised species of the genus; (iii) to perform molecular characterisation of these populations using D2–D3 expansion segments of 28S rRNA, ITS rRNA, partial 18S rRNA, and *COI* gene sequences; and (iv) to infer the phylogenetic relationships of the identified species within the context of currently available *Paralongidorus* and *Longidorus* molecular datasets.

## Materials and methods

### Sampling and nematode identification

Nematode surveys were conducted across forested areas of northern Spain, encompassing a range of woodland types dominated by species such as downy oak (*Quercus pubescens* Willd.), chestnut (*Castanea sativa* Mill.), coast redwood (*Sequoia sempervirens* (D.Don) Endl.), holly (*Ilex aquifolium* L.), and common beech (*Fagus sylvatica* L.). Sampling was carried out during the early summer of 2024 and 2025, and comprised 20 sampling sites widely distributed across the Cantabria and Navarra provinces in northern Spain.

At each location, soil samples were collected from the rhizosphere of host plants using a hoe, targeting the upper 50 cm of soil. From each bulk sample, a 500 cm^3^ subsample was processed for nematode extraction using the sieving method described by Flegg [25]. Extracted *Paralongidorus* specimens were heat-killed, fixed in a solution of 4% formaldehyde and 1% propionic acid, and processed into pure glycerine using Seinhorst’s method [26].

Light micrographs and measurements of the two collected *Paralongidorus* populations were obtained using a Leica DM6 compound microscope equipped with a Leica DFC7000 T digital camera (Wetzlar, Germany). Morphological assessments included key diagnostic characteristics such as de Man indices, body length, odontostyle length, lip region width, tail length and shape, and guiding ring distance from the anterior end [27]. Specimens were mounted in glycerine for detailed examination.

Nematodes were identified to species level using an integrative approach combining morphological analyses of females and J1–J4 juveniles [27] with molecular analyses to ensure precise and reliable species delineation [28]. All specimens were mounted on microscope slides in glycerine.

### DNA extraction, PCR and sequencing

Both *Paralongidorus* populations collected in this study were subjected to molecular characterisation. In each case, DNA extraction was performed from individual specimens, ensuring that all molecular markers analysed originated from the same single DNA-extracted specimen per PCR tube, thereby eliminating potential cross-contamination. To prevent misidentification in cases where potential multiple *Longidorus* and *Paralongidorus* populations could co-occurred within the same soil sample, individual nematodes were temporarily placed in a drop of 1 M NaCl containing glass beads. This procedure minimised physical damage while allowing confirmation of specimen identity within the target population.

DNA extraction from nematodes followed the protocol described by Subbotin et al. [29]. Briefly, individual specimens were cut with a scalpel in a 20 µL drop of PCR buffer (ThermoPol®, New England Biolabs, USA), to which 2 μL of proteinase K (600 μg/mL) was added. Tubes were frozen at − 80 °C for 15 minutes, followed by sequential incubations at 65 °C for 1 hour and 95 °C for 10 minutes. Samples were then centrifuged at 16,000× g for 1 minute and stored at − 20 °C until use in PCR.

The D2–D3 expansion segments of the 28S rRNA gene were amplified using primers D2Ab (5’-ACAAGTACCGTGAGGGAAAGTTG-3’) and D3B (5’-TCGGAAGGAACCAGCTACTA-3’) [30]. The ITS region was amplified using the forward primer 18S (5′-TTGATTACGTCCCTGCCCTTT-3′) and the reverse primer 26S (5′-TTTCACTCGCCGTTACTAAGG-3′) [31]. Partial 18S rRNA gene was amplified using primers 988F (5′-CTCAAAGATTAAGCCATGC-3′), 1912 R (5′-TTTACGGTCAGAACTAGGG-3′), 1813F (5′-CTGCGTGAGAGGTGAAAT-3′), and 2646 R (5′-GCTACCTTGTTACGACTTTT-3′) [32]. The mitochondrial *COI* gene fragment was amplified using primers COIF (5′-GATTTTTTGGKCATCCWGARG-3′) and COIR (5′-CWACATAATAAGTATCATG-3′) following the protocol of Lazarova et al. [33].

All PCR assays were performed under the conditions described by Archidona-Yuste et al. [34]. Amplified PCR products were subsequently purified using ExoSAP-IT (Affymetrix, USB Products) and sequenced on a 3130XL Genetic Analyser (Applied Biosystems, Foster City, CA, USA) using the BigDye Terminator Sequencing v0.3.1 Cycle Sequencing Kit (Applied Biosystems). Sequencing was carried out at the StabVida facility (Costa da Caparica, Portugal).

Newly obtained sequences were submitted to the National Center for Biotechnology Information (NCBI) under accession numbers listed in Table 1 and associated phylogenetic trees. Sequence chromatograms for all four markers (D2–D3 expansion segments of 28S, ITS, 18S rRNA, and *COI* mtDNA) were analysed using DNASTAR LASERGENE SeqMan v0.7.1.0. DNA sequences were compared using BLAST [35] to other sequences from each molecular marker in National Center for Biotechnology Information (NCBI).

**Table 1:** *Paralongidorus* specimens analysed and sequenced in the present study, collected from forested habitats in northern Spain. Sampling localities include soba (Cantabria) and Elizondo (Navarra), with details of specimen counts, amplified gene regions, and associated GenBank accession numbers

Species	Sample code	Location	Associated plant	**Nematode density per 500 cm**^**3**^**of soil**^**a**^	Ribosomal and mitochondrial markers			
					28S D2–D3	ITS	18S	COI
*Paralongidorus cantabronavarrus* sp. nov.	CNT1	Soba, Cantabria (Spain)	*Fagus sylvatica* L. (common beech)	10	PX369417- PX369422	PX369429- PX369432	PX369436- PX369437	PX369900- PX369905
*Paralongidorus cantabronavarrus* sp. nov.	EL10	Elizondo, Navarra (Spain)	*Fagus sylvatica* L. (common beech)	37	PX369423- PX369428	PX369433- PX369435	PX369438	PX369906- PX369907

^a^*Paralongidorus* nematode populations collected per sampling site and extracted using the sieving method described by Flegg (1967) [23]

### Phylogenetic analyses

Phylogenetic reconstruction was performed using D2–D3 expansion segments of the 28S rRNA, ITS rRNA, partial 18S rRNA, and mitochondrial *COI* gene sequences from the two collected *Paralongidorus* populations, along with available *Longidorus* and *Paralongidorus* accessions from NCBI. Outgroup taxa were selected based on previously published studies [32, 36–39] to ensure comprehensive molecular variation within the analysed sequences [40].

Multiple sequence alignments were conducted using the FFT-NS-2 algorithm implemented in MAFFT v0.7.450 [41]. Alignments were visualised and manually edited in BioEdit v0.7.2.5 [42], where poorly aligned positions were trimmed using a light filtering strategy (up to 20% of alignment positions). This approach minimises the impact on tree accuracy while reducing computation time, consistent with the recommendations of Tan et al., 2015 [43], given that automated filtering methods often degrade single-gene phylogenetic inference. Phylogenetic analyses were performed using Bayesian inference (BI) in MrBayes v0.3.1.2 [44]. The best-fit model of DNA evolution was determined using JModelTest v0.2.1.7 [45] under the Akaike information criterion (AIC). The selected models, along with estimated base frequencies, the proportion of invariable sites, gamma distribution shape parameters, and substitution rates, were implemented in MrBayes for phylogenetic reconstruction. The following evolutionary models were applied: D2–D3 expansion segments of 28S rRNA: Symmetrical model with invariable sites and gamma distribution (SYM + I + G); ITS region: General Time-Reversible model with a gamma distribution (GTR + G); partial 18S rRNA and partial *COI* gene: General Time-Reversible model with invariable sites and gamma distribution (GTR + I + G). All Bayesian analyses were run separately for each dataset using four chains over 10 × 10^6^ generations, with Markov chain sampling at intervals of 100 generations. Two independent runs were conducted per dataset. After discarding the first 30% of samples as burn-in and assessing convergence, the remaining trees were retained for further analysis. A 50% majority-rule consensus tree was generated, with posterior probabilities (PP) assigned to each relevant clade. Phylogenetic trees were visualised using FigTree v0.1.4.4 [46]. All sequence alignments and original tree files generated during the phylogenetic analyses are publicly available at the Zenodo repository at https://doi.org/10.5281/zenodo0.17144705.

## Results

**Fig. 1:**
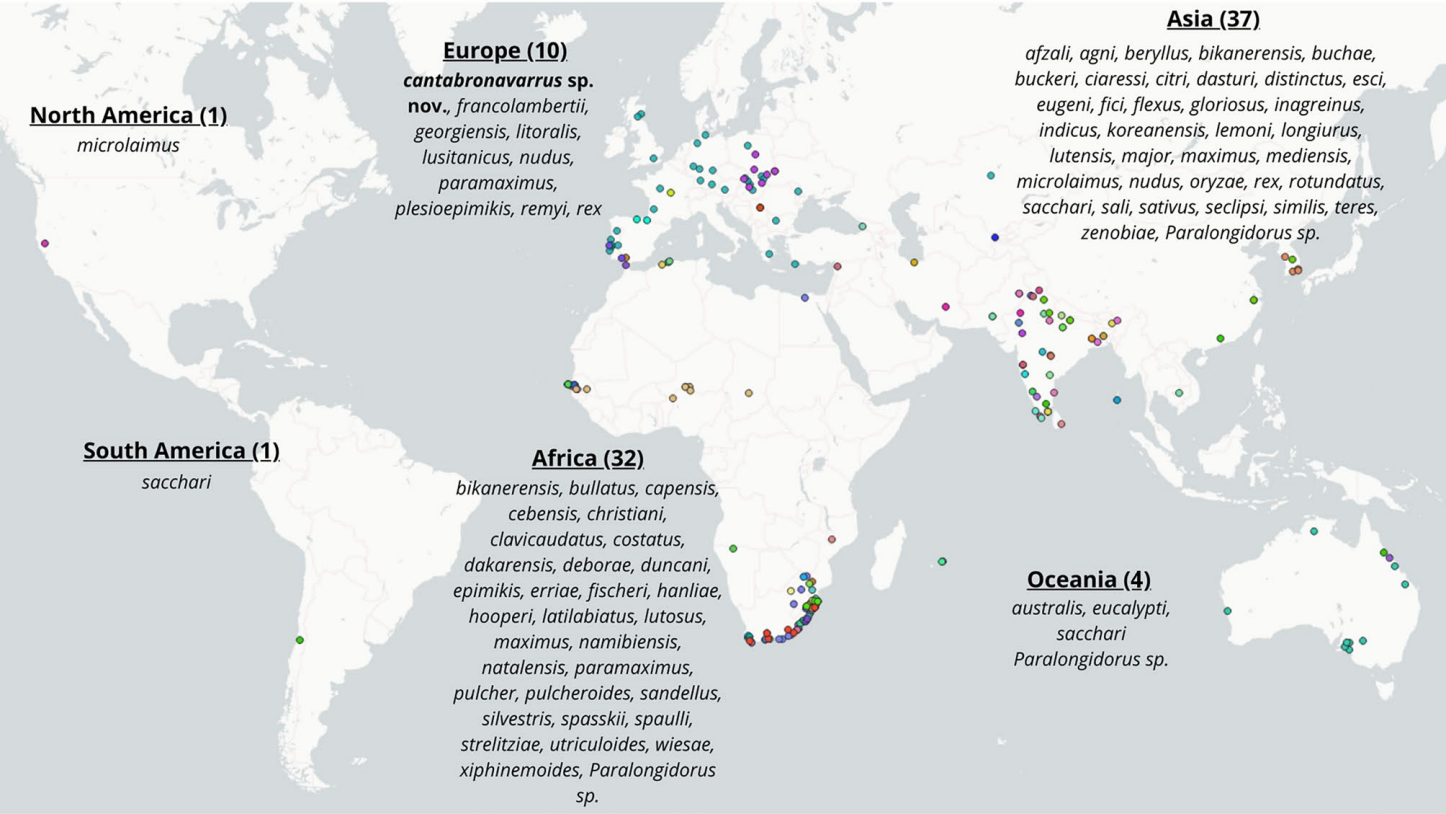
Global distribution of needle nematode species within the genus *Paralongidorus* Siddiqi, Hooper and Khan, 1963 [1]. Each coloured dot on the map denotes a distinct species, with further details on species occurrences across continents provided in Table S1

Two out of 20 soil samples (10% overall prevalence) collected from forested areas in northern Spain tested positive for *Paralongidorus* spp. (Fig. 1, Table 1). A total of 47 nematodes from both populations were examined, including 14 adult females, two adult males, and 19 juveniles representing the first to fourth developmental stages. Of these, one female and 12 juvenile specimens were used for molecular analyses. Detailed morphological, morphometric, and molecular analyses of these two populations revealed a single, novel, previously undescribed species of the genus, *Paralongidorus cantabronavarrus* sp. nov., which is formally described herein.

Based on the literature review conducted in this study, members of the genus *Paralongidorus* have been reported from every continent, except Antarctica (Fig. 1, Table S1). The highest species richness is observed in Asia, with 37 species recorded across multiple countries, the majority of which (30 species) are reported from India (Fig. 1, Table S1). Africa follows with 32 species, notably including 23 reported from South Africa (Fig. 1, Table S1). In contrast, other continents exhibit markedly lower diversity: Europe contains ten species, including six recorded species from the Iberian Peninsula (Fig. 1, Table S1); Oceania hosts four species; and North and South America each harbour only a single species, recorded in California [47] and Chile [48], respectively (Fig. 1, see Table S1 for a complete list of global records).

### Taxonomic revision

Escuer and Arias [18] recorded a total of 70 nominal species within the genus *Paralongidorus*, including Longidoroides Khan, Chawla and Saha, 1976 [8], Siddiqia Khan, Chawla and Saha, 1976 [8] and *Inagreius* Khan, 1982 [12], which were subsequently treated as junior synonyms of *Paralongidorus*. Since then, nine additional species have been described from Iran, Korea, Portugal, Senegal, Serbia, South Korea, Spain and Syria (see nominal list below). Furthermore, based on detailed examinations of paratypes and new SEM studies, several species have been reassigned to the genus *Longidorus* by Roca [48], Decraemer and Coomans [6] and Clavero-Camacho et al. [19]. These include: *L. boshi* (Khan et al. 1972) Decraemer and Coomans, 2007 [6, 7], *L. cedari* (Khan et al. 1976) Decraemer and Coomans, 2007 [6, 49], *L. iberis* (Escuer and Arias, 1997) Clavero-Camacho et al. 2022 [18, 19], *L. iranicus* (= *P. serbicus, = L. moesicus*)(Krnjaic et al. 2002) Roca, 2006 [50, 51], *L. milanis* (Krnjaic et al. 2002) Roca, 2006 [50, 51], *L. monegrensis* (Escuer and Arias, 1997) Decraemer and Coomans, 2007 [6, 18], and *L. spiralis* (Khan et al. 1972) Decraemer and Coomans, 2007 [6, 7]. These taxonomic revisions bring the current total to 75 valid nominal species of *Paralongidorus*, in addition to the novel species described in this study (see nominal list below). Accordingly, the polytomous key has been revised and updated to reflect these changes (Table S2).

## Taxonomy

**Phylum:** Nematoda Rudolphi, 1808 [52]

**Class:** Enoplea Inglis, 1983 [53]

**Order:** Dorylaimida Pearse, 1942 [54]

**Suborder:** Dorylaimina Pearse, 1936 [55]

**Superfamily:** Longidoroidea Khan and Ahmad, 1975 [56]

**Family:** Longidoridae Thorne, 1935 [7]

**Genus:***Paralongidorus* Siddiqi, Hooper and Khan, 1963 [1]

syn. *Longidoroides* Khan, Chawla and Saha, 1976 [8]

syn. *Siddiqia* Khan, Chawla and Saha, 1976 [8]

syn. *Inagreius* Khan, 1982 [12]

syn. *Paralongidorus* (*Paralongidorus*) Hunt, 1993 [15]

syn. *Paralongidorus* (*Siddiqia*) Hunt, 1993 [15]

### Updated list of valid nominal species

Type species: *Paralongidorus sali* Siddiqi, Khan and Hooper, 1963 [1]

Other species:

1. * P. afzali* (Khan, 1964) Siddiqi and Husain 1965 [57, 58] syn. *Longidorus afzali* Khan, 1964 [58]
2. *P. agni* Sharma and Edward, 1985 [59]
3. *P australis* Stirling and McCulloch, 1984 [60]
4. *P. beryllus* Siddiqi and Husain, 1965 [57]
5. *P. bikanerensis* (Lal and Mathur, 1987) Siddiqi, Baujard and Mounport, 1993 [3, 61] syn. *Longidoroides bikanerensis* Lal and Mathur, 1987 [61]

6. *P. buchae* Lamberti, Roca and Chinappen, 1985 [62]
7. *P. buckeri* Sharma and Edward, 1985 [59]
8. *P. bullatus* Sharma and Siddiqi, 1990 [63]
9. ***Paralongidorus cantabronavarrus *sp. nov.** García-Velázquez, Salazar-García, Cantalapiedra-Navarrete, Criado-Navarro, Palomares-Rius, Castillo and Archidona-Yuste, 2026
10. *P. capensis* Heyns, 1966 [11] syn. *Siddiqia natalensis* Jacobs and Heyns, 1982 [64]

11. *P. cebensis* Heyns and Coomans, 1989 [65]
12. *P. christiani* Liebenberg, Heyns, Swart, 1993 [66]
13. *P. ciaressi* Dhanam and Jairajpuri, 1997 [67]
14. *P. citri* (Siddiqi, 1959) Siddiqi, Hooper and Khan, 1963 [1, 68], syn. *Xiphinema citri* Siddiqi, 1959 [68], syn. *Longidorus citri* (Siddiqi, 1959) Thorne, 1961 [68, 69], syn. *Paralongidorus droseri* (Sukul, 1972) Thorne, 1961 [69, 70]

15. *P. clavicaudatus* (Jacobs and Heyns, 1982) Hunt, 1993 [15, 64]
16. *P. costatus* (Jacobs and Heyns,1987) Siddiqi, Baujard, and Mounport, 1993 [3, 71], syn. *Longidoroides costatus* Jacobs and Heyns, 1987 [71]
17. *P. dakarensis* Faye and Mounport, 2007 [72]
18. *P. dasturi* (Ganguly, Patil and Khan, 1981) Luc and Doucet, 1984 [13, 73] syn. *Siddiqia dasturi* Ganguly, Patil and Khan, 1981 [73]

19. *P. deborae* (Jacobs and Heyns, 1982) Luc and Doucet, 1984 [13, 64] syn. *Siddiqia deborae* Jacobs and Heyns,1982 [64]
20. *P. distinctus* Baqri and Jairajpuri, 1981 [74]
21. *P. duncani* Siddiqi, Baujard and Mounport, 1993 [3]
22. *P. epimikis* Dalmasso, 1969 [75]
23. *P. erriae* Heyns, 1965 [76]
24. *P. esci* Khan, Chawla and Saha, 1976 [8]
25. *P. eucalypti* Fisher, 1964 [77]
26. *P. eugeni* (Khan, 1986) Hunt, 1993 [15, 78] syn*. Inagreius eugeni* Khan, 1986 [78]

27. *P. fici* Edward, Misra and Singh, 1964 [79]
28. *P. fischeri* Heyns, 1972 [80]
29. *P. flexus* Khan, Seshadri, Weischer and Mathen, 1971 [81]
30. *P. francolambertii* Barsi and De Luca, 2017 [22]
31. *P. georgiensis* (Tulaganov, 1937) Siddiqi, 1964 [82, 83]
32. *P. gloriosus* (Khan, 1982) Hunt, 1993 [12, 15] syn. *Inagreius gloriosus* Khan, 1982 [12]

33. *P. halepensis* Lamberti, Molinari, De Luca, Agostinelli and Di Vito, 1999 [84]
34. *P. hanliae* Liebenberg, Heyns and Swart, 1993b [66]
35. *P. hooperi* Heyns, 1966 [11]
36. *P. inagreinus* (Chawla and Samathanam, 1981) Luc and Doucet, 1984 [13, 85] syn. *Siddiqia inagreina* Chawla and Samathanam, 1981 [85]

37. *P. indicus* (Phukan and Sanwal, 1983) Luc and Doucet, 1984 [13, 86] syn. *Siddiqia indicus* Phukan and Sanwal, 1983 [86]
38. *P. iranicus* Pedram, Pourjam, Namjou, Atighi, Cantalapiedra-Navarrete, Liébanas, Palomares-Rius and Castillo, 2012 [21]
39. *P. koreanensis* Mwamula, Decraemer, Kim, Ko, Na, Kim and Lee, 2020 [5]
40. *P. latilabiatus* (Jacobs and Heyns, 1982) Siddiqi, Baujard, and Mounport, 1993 [3, 64] syn. *Longidoroides latilabiatus* Jacobs and Heyns, 1982 [64]

41. *P. lemoni* Nasira, Shahina, Firoza and Maqbool, 1993 [87]
42. *P. litoralis* Palomares-Rius, Subbotin, Landa, Vovlas and Castillo, 2008 [20]
43. *P. longiurus* (Chawla and Samathanam, 1981) Siddiqi, Baujard and Mounport, 1993 [3, 85] syn. *Longidoroides longiurus* Chawla and Samathanam, 1981 [85]

44. *P. lusitanicus* Gutiérrez-Gutiérrez, Mota, Castillo, Santos and Palomares-Rius, 2018 [23]
45. *P. lutensis* Hunt and Rahman, 1991 [88]
46. *P. lutosus* (Heyns, 1965) Aboul-Eid, 1970 [76, 89] syn. *Longidorus lutosus* Heyns, 1965 [76]

47. *P. major* Verma, 1973 [90]
48. *P. maximus* (Bütschli, 1874) Siddiqi, 1964 [83, 91] syn. *Dorylaimus maximus* Bütschli, 1874 [91] syn. *Longidorus maximus* (Bütschli, 1874) Thorne and Swanger, 1936 [91, 92]

49. *P. mediensis* (Ganguly, Patil and Khan, 1981) Luc and Doucet, 1984 [13, 73] syn. *Siddiqia mediensis* Ganguly, Patil and Khan,1981 [73]
50. *P. microlaimus* Siddiqi, 1964 [83]
51. *P. namibiensis* Jacobs and Heyns, 1987 [71]
52. *P. nudus* (Kirjanova, 1951) Lamberti, 1975 [93, 94] syn. *Longidorus nudus* Kirjanova, 1951 [93]

53. *P. oryzae* Verma, 1973 [90]
54. *P. paramaximus* Heyns, 1965 [76]
55. *P. pini* (Jacobs and Heyns, 1987) Siddiqi, Baujard and Mounport, 1993 [3, 71] syn. *Longidoroides pini* Jacobs and Heyns, 1987 [71]

56. *P. plesioepimikis* Palomares-Rius, Cantalapiedra-Navarrete, Gutiérrez-Gutiérrez, Liébanas and Castillo, 2013 [4]
57. *P. pulcher* (Jacobs and Heyns, 1982) Siddiqi, Baujard and Mounport, 1993 [3, 64] syn. *Longidoroides pulcher* Jacobs and Heyns, 1982 [64]

58. *P. pulcheroides* (Jacobs and Heyns, 1987) Siddiqi, Baujard and Mounport, 1993 [3] syn. *Longidoroides pulcheroides* Jacobs and Heyns, 1987 [71]
59. *P. remyi* (Altherr, 1963) Siddiqi and Husain, 1965 [57, 95] syn. *Longidorus remyi* Altherr, 1963 [95]
60. *P. rex* Andrássy, 1986 [96]
61. *P. rotundatus* Khan, 1987 [97]
62. *P. sacchari* Siddiqi, Khan and Hooper, 1963 [1]
63. *P. sali* Siddiqi, Khan and Hooper, 1963 [1]
64. *P. sandellus* (Heyns, 1966) Coomans (1985) [11, 14] syn. *Xiphinema sandellum* Heyns, 1966 [11], syn. *Longidorus sandellus* (Heyns, 1966) Khan, Chawla and Saha, 1976 [8, 11] syn. *Brevinema sandellum* (Heyns, 1966) Chaves and Coomans, 1984 [11, 98]

65. *P. sativus* (Soni and Nama, 1983) Escuer and Arias, 1997 [18, 99] syn. *Longidoroides sativus* Soni and Nama, 1983 [99] syn. *Siddiqia seclipsi* Khan, Singh and Singh, 1981 [100] syn. *Longidoroides seclipsi* (Khan, Singh and Singh, 1981) Luc and Doucet, 1984 [13, 100]
66. *P. seclipsi* (Khan, Singh and Singh, 1981) Jana and Baqri, 1984 [100, 101]
67. *P. silvestris* Faye and Mounport, 2010 [102]
68. *P. similis* Khan, Chawla and Prasad, 1972 [103]
69. *P. spasskii* Heyns, 1972 [80]
70. *P spaulli* (Jacobs and Heyns, 1982) Luc and Doucet, 1984 [13, 64] syn. *Siddiqia spaulli* Jacob and Heyns, 1982 [64]

71. *P. strelitziae* (Heyns, 1966) Aboul-Eid, 1970 [11, 89] syn. *Longidorus strelitziae* Heyns, 1966 [11] syn. *Longidoroides strelitziae* (Heyns, 1966) Khan, Chawla and Saha, 1976 [8, 11]
72. *P. teres* (Khan, 1987) Hunt, 1993 [15, 97] syn. *Inagreius teres* Khan, 1987 [97] syn. *Longidoroides teres* (Khan, 1987) Jairajpuri and Ahmad, 1992 [27, 97]
73. *P. utriculoides* (Corbett, 1964) Siddiqi and Husain, 1965 [57, 104] syn. *Longidorus utriculoides* Corbett, 1964 [104]
74. *P. wiesae* (Heyns, 1994) Escuer and Arias, 1997 [18, 105] syn. *Longidoroides wiesae* Heyns, 1994 [105]
75. *P. xiphinemoides* Heyns, 1965 [76]
76. *P. zenobiae* Hunt and Rahman, 1991 [88]

**Fig. 2:**
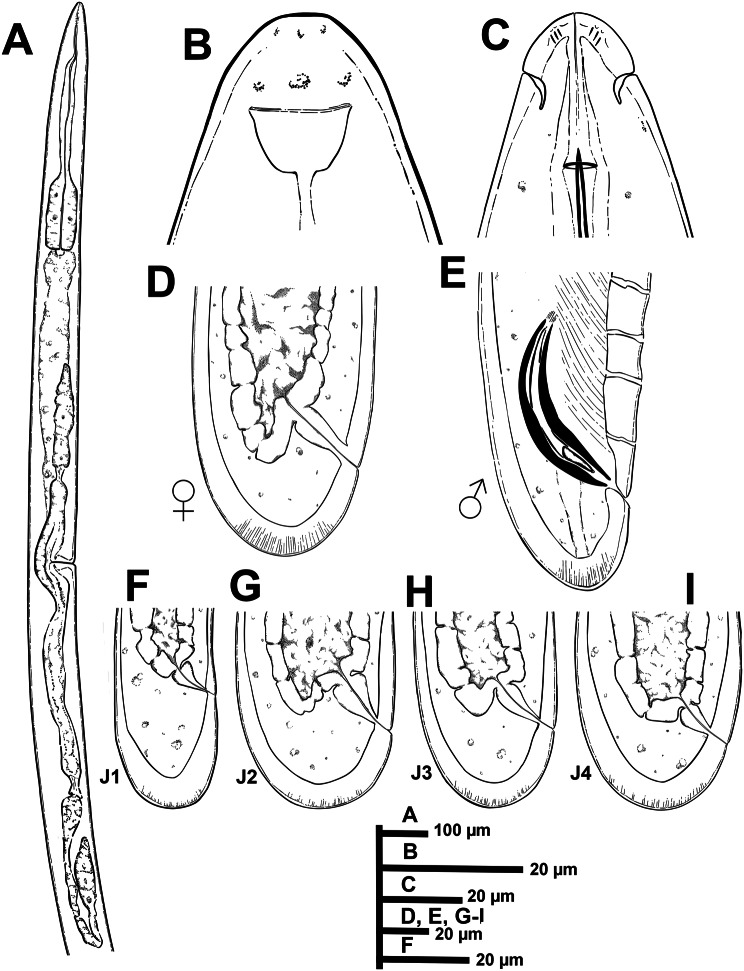
Line drawings of *Paralongidorus cantabronavarrus* sp. nov. holotype. **A** Female anterior body region, showing reproductive system; **B**, **C** lip region showing amphidial fovea in lateral and dorsal view; **D** female tail; **E** male tail; **F**-**I** tail of 1st, 2nd, 3rd, and 4th stage juveniles (J1, J2, J3 and J4), respectively

**Fig. 3:**
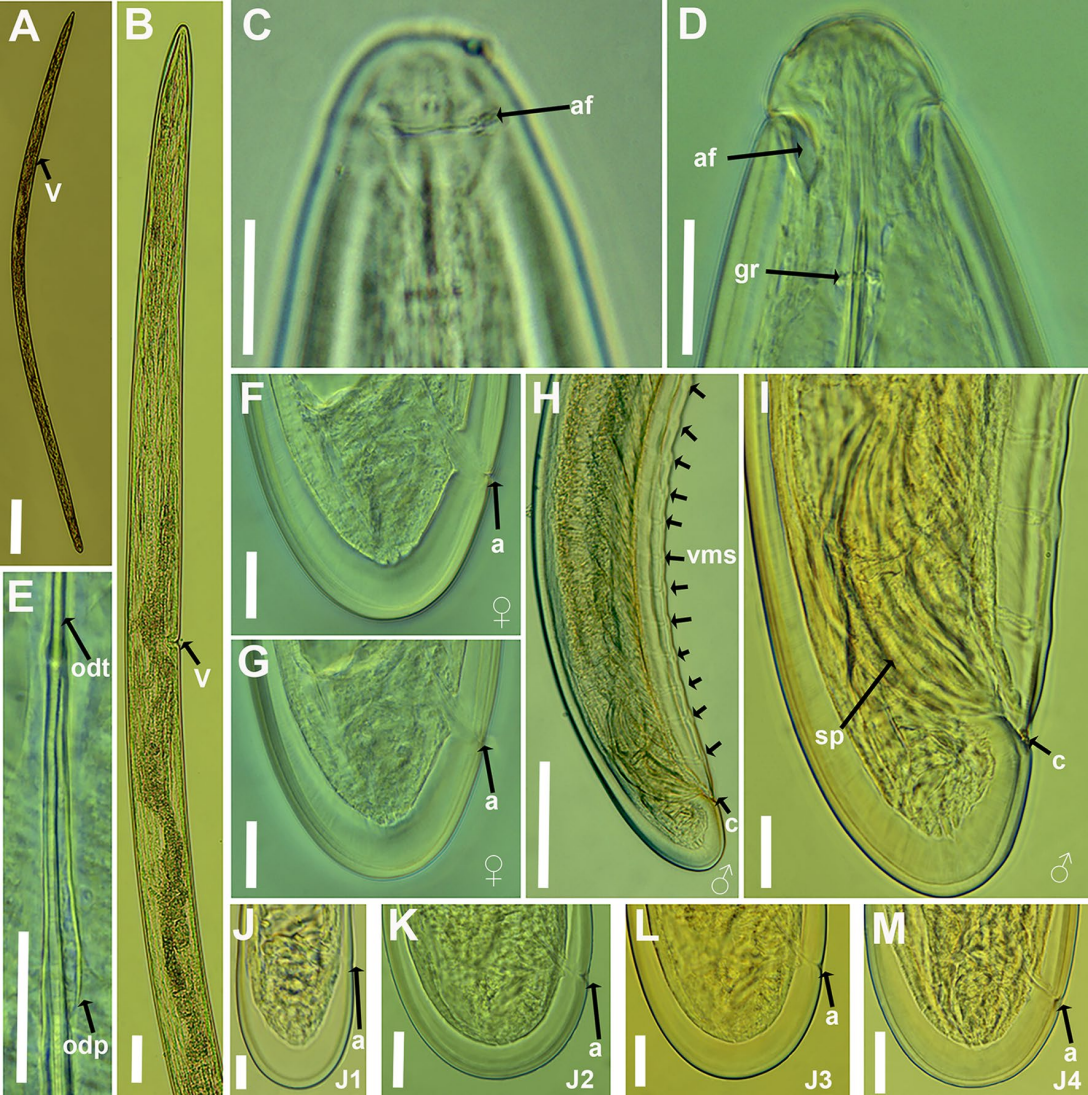
Light microphotographs of *Paralongidorus cantabronavarrus* sp. nov. **A** Entire female; **B** anterior body region of female showing vulval position; **C** lateral view of female lip region with amphidial aperture indicated (arrow); **D** dorsal view of female lip region with amphidial fovea indicated (arrow); **E** detail of odontostyle and odontophore junction; **F**, **G** female tail region; **H**-**I** male tail region showing ventromedian supplements and spicules (arrowed); **J**-**M** tail regions of first to fourth juvenile stages (J1–J4). Abbreviations: a = anus; af = amphidial fovea; c = cloaca; gr = guiding ring; odt = odontostyle; odp = odontophore; V = vulva; vms = ventromedian supplements. Scale bars a = 500 µm; B, H = 100 µm; C-G, I-M = 20 µm

**Fig. 4:**
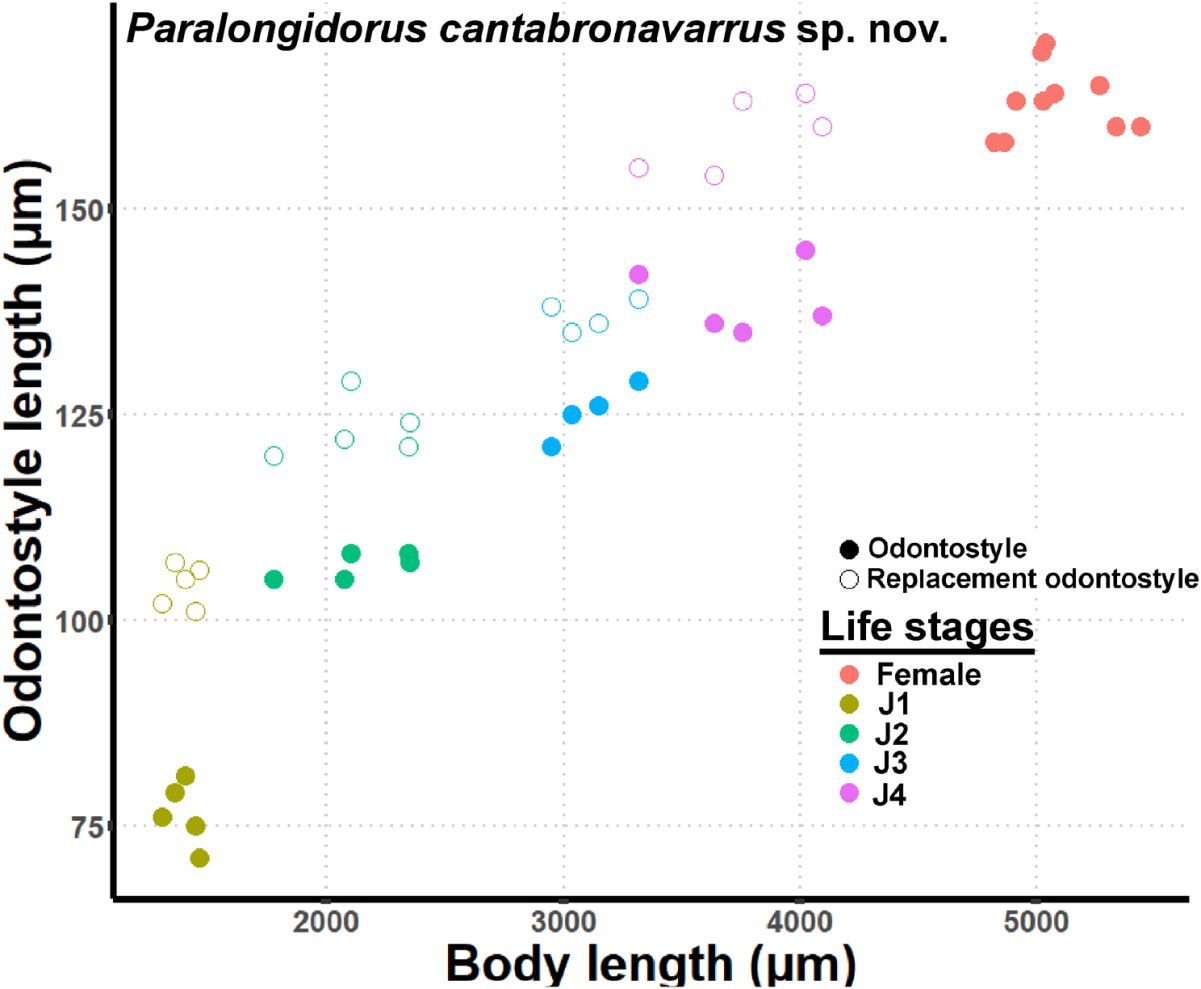
Relationships of body length to length of functional and replacement odontostyle in all developmental stages from first-stage juvenile (J1) to mature females of *Paralongidorus cantabronavarrus* sp. nov

***Paralongidorus cantabronavarrus *sp. nov.** (Figs. 2–4; Table 2)

**Table 2:** Morphometric data for *Paralongidorus cantabronavarrus* sp. nov. (type and voucher specimens) collected from common beech (*Fagus sylvatica* L.) at Soba, Cantabria province and Elizondo, Navarra province, northern Spain. All measurements are in µm, except for body length in mm. Values are expressed as mean ± standard deviation (range)

		Paratypes (Soba, Cantabria province)						Voucher specimens (Elizondo, Navarra province)	
Character/ratio^1^	Holotype	Females	Male	J1	J2	J3	J4	Females	Male
n	1	10	1	5	5	4	5	3	1
L (mm)	5.074	5.077 ± 0.256	4.741	1.392 ± 0.643	2.128 ± 0.237	3.112 ± 0.158	3.763 ± 0.313	5.113 ± 0.295	4.607
		(4.82–5.67)		(1.30–1.46)	(1.78–2.35)	(2.95–3.32)	(3.32–4.09)	(4.87–5.44)	
a	49.3	46.6 ± 2.7	49.4	43.3 ± 2.8	40.7 ± 4.0	44.8 ± 3.5	42.1 ± 2.8	43.5 ± 2.9	49.0
		(42.3–50.0)		(39.5–46.7)	(36.3–45.2)	(40.4–49.0)	(38.7–45.5)	(40.2–45.8)	
b	9.9	9.4 ± 0.6	8.0	4.7 ± 0.4	5.6 ± 1.1	7.4 ± 0.5	8.3 ± 0.7	8.9 ± 0.7	7.6
		(8.5–10.1)		(4.3–5.1)	(4.7–7.5)	(7.0–7.8)	(7.6–9.1)	(8.4–9.6)	
c	134.7	125.6 ± 7.3	107.8	51.2 ± 2.7	63.9 ± 8.8	85.4 ± 5.7	104.8 ± 13.8	120.1 ± 7.1	124.5
		(111.6–136.4)		(48.4–55.6)	(54.5–78.2)	(77.6–89.9)	(85.0–120.3)	112.0–124.7)	
c’	0.7	0.6 ± 0.1	0.8	1.0 ± 0.1	0.8 ± 0.1	0.8 ± 0.05	0.7 ± 0.1	0.6 ± 0.04	0.7
		(0.5–0.7)		(1.0–1.1)	(0.6–0.9)	(0.7–0.8)	(0.6–0.8)	(0.6–0.7)	
d	2.0	2.1 ± 0.1	2.2	2.1 ± 0.2	1.7 ± 0.05	1.7 ± 0.1	1.8 ± 0.04	2.1 ± 0.1	1.9
		(2.0–2.1)		(1.8–2.3)	(1.7–1.8)	(1.6–1.8)	(1.7–1.8)	(2.0–2.2)	
d’	2.2	2.3 ± 0.1	2.3	2.0 ± 0.1	1.9 ± 0.1	2.0 ± 0.2	2.0 ± 0.1	2.5 ± 0.1	2.2
		(2.2–2.4)		(1.9–2.1)	(1.8–2.1)	(1.9–2.3)	(1.9–2.2)	(2.3–2.5)	
V or T	28.6	27.9 ± 1.0	55.3	-	-	-	-	27.3 ± 0.8	42.8
		(26.2–28.8)						(26.7–28.2)	
G_1_	8.9	9.0 ± 0.4	-	-	-	-	-	9.4 ± 0.5	-
		(8.3–9.7)						(8.8–9.7)	
G_2_	15.0	15.2 ± 1.2	-	-	-	-	-	16.1 ± 0.8	-
		(12.8–16.7)						(15.3–16.8)	
Odontostyle	164.0	162.8 ± 3.3	151.0	76.4 ± 3.8	106.6 ± 1.5	125.3 ± 3.3	139.0 ± 4.3	162.7 ± 6.4	162.0
		(158.0–169.0)		(71.0–81.0)	(105.0–108.0)	(121.0–129.0)	(135.0–145.0)	(158.0–170.0)	
Odontophore	50.0	51.6 ± 1.9	51.0	42.4 ± 1.1	48.8 ± 1.3	54.0 ± 2.2	54.6 ± 1.7	54.3 ± 2.1	55.0
		(50.0–55.0)		(41.0–44.0)	(47.0–50.0)	(51.0–56.0)	(52.0–56.0)	(52.0–56.0)	
Total stylet	214.0	214.5 ± 3.9	202.0	118.8 ± 4.3	155.4 ± 1.7	179.3 ± 4.3	193.6 ± 5.0	217.0 ± 8.2	217.0
		(210.0–224.0)		(114.0–125.0)	(153.0–157.0)	(175.0–185.0)	(189.0–200.0)	(210.0–226.0)	
Replacement odontostyle	-	-	-	104.2 ± 2.6	123.2 ± 3.6	137.0 ± 1.8	159.2 ± 4.5	-	-
				(101.0–107.0)	(120.0–129.0)	(135.0–139.0)	(154.0–164.0)		
Lip region diam.	17.0	17.4 ± 0.6	17	9.3 ± 0.7	14.5 ± 0.5	15.3 ± 0.5	17.1 ± 0.9	17.0 ± 1.0	18
		(16.5–18.0)		(8.5–10.0)	(14.0–15.0)	(15.0–16.0)	(16.0–18.0)	(16.0–18.0)	
Oral aperture to guiding ring	36.0	35.7 ± 1.4	37.0	19.5 ± 1.9	25.0 ± 1.2	26.6 ± 1.8	30.6 ± 1.1	35.3 ± 2.1	34.0
		(33.0–37.0)		(17.5–22.0)	(24.0–27.0)	(24.0–28.0)	(29.0–32.0)	(33.0–37.0)	
Max. body diam.	103.0	109.2 ± 6.1	96.0	32.2 ± 1.5	52.4 ± 5.1	69.8 ± 5.7	89.6 ± 9.1	117.7 ± 6.7	94.0
		(100.0–119.0)		(30.0–34.0)	(44.0–57.0)	(62.0–75.0)	(80.0–100.0)	(110.0–122.0)	
Tail length	41.0	40.5 ± 2.9	44.0	27.2 ± 1.3	33.6 ± 4.4	36.5 ± 2.4	36.2 ± 3.6	42.7 ± 3.2	37.0
		(36.0–45.0)		(26.0–29.0)	(28.0–38.0)	(34.0–39.0)	(33.0–41.0)	(39.0–45.0)	
Tail hyaline region	14.0	14.0 ± 1.2	10.0	8.2 ± 0.8	10.4 ± 1.9	12.0 ± 0.8	12.2 ± 1.5	15.0 ± 2.0	12.0
		(13.0–17.0)		(7.0–9.0)	(9.0–13.0)	(11.0–13.0)	(10.0–14.0)	(13.0–17.0)	
Spicules	-	-	102.0	-	-	-	-	-	94.0

^1^ Abbreviations as defined in Jairajpuri & Ahmad [25]. a, body length/maximum body width; b, body length/pharyngeal length; c, body length/tail length; c’, tail length/body width at anus; V (distance from anterior end to vulva/body length) ×100; d = anterior to guiding ring/body diam. at lip region; d’ = body diam. at guiding ring/body diam. at lip region

#### Zoobank

urn:lsid:zoobank.org:act:5CB1D638-1190–4461-921A-8796DC9C53F4

#### Holotype

Adult female collected from a soil sample from the rhizosphere of common oak (*Fagus sylvatica* L.), in Soba, Cantabria, northern Spain (43°11’57.78” N latitude, 3°38’17.43” W longitude, 1090 m a.s.l.) by P. Castillo, mounted in pure glycerine and deposited in the Nematode Collection of the Institute for Sustainable Agriculture, CSIC, Córdoba, Spain (slide number CNT1_1).

#### Paratypes

Ten females, one male, and five specimens from each juvenile-stage (J1–J4), except four from J3, were collected as paratypes simultaneously with the holotype from the type locality by P. Castillo. All specimens were mounted in pure glycerine and deposited in the Nematode Collection of the Institute for Sustainable Agriculture, CSIC, Córdoba, Spain (slide numbers CNT1_3 to CNT_10). Additionally, one female paratype was deposited on slide number *T*-845t in the USDA Nematode Collection, Beltsville, MD, USA.

#### Voucher specimens

A second population was recovered from the rhizosphere of common beech (*Fagus sylvatica* L.) at Elizondo, Navarra, northern Spain (43°04’00.75” N latitude, 1°36’15.11” W longitude, 899 m a.s.l.), comprising three females and one male.

#### Etymology

The species epithet *cantabronavarrus* is a Latinised adjective meaning “from Cantabria and Navarra”, referring to the regions in northern Spain where the species was detected. It denotes either a geographical association with these areas or a connection to their ancient inhabitants.

#### Diagnosis and relationships

*Paralongidorus cantabronavarrus* sp. nov. is an amphimictic species characterised by a moderately long body (4.8–5.7 mm); a conoid-rounded lip region, continuous with the rest of the body (16.5–18.0 μm wide); a large stirrup-shaped amphidial fovea; a long odontostyle (158–169 μm); an anterior position of the vulva (V = 26–29), and a female tail that is conoid-rounded with broadly rounded terminus. Based on the polytomous key by Escuer and Arias [18], and considering the morphology of the amphidial fovea, lip region, female tail, and odontostyle length, *P. cantabronavarrus* sp. nov. is closely related to *P. australis*, *P. distinctus*, *P. esci*, *P. sacchari*, and *P. sali*. However, it can be distinguished from these species by a unique combination of morphological and morphometric characters, most notably the markedly anterior position of the vulva.

*Paralongidorus cantabronavarrus* sp. nov. differs from *P. australis* by having a shorter body (4.8–5.7 vs. 7.6–10.6 mm), a shorter distance from the guiding ring to the anterior end (33–37 vs. 58–70 μm), an anterior position of the vulva (V = 26–29 vs. 46–56), a longer female tail (36–45 vs. 21–35 μm), and shorter spicules (94–102 vs. 112–134 µm). It can be distinguished from *P. distinctus* by the absence of males in the latter, a shorter body (4.8–5.7 vs. 7.35 mm), differences in lip region shape and width (conoid-rounded, 16.5–18.0 µm vs. subtruncate, 12 µm), a shorter distance from the guiding ring to the anterior end (33–37 vs. 45 μm), a longer female tail (36–45 vs. 33 μm), and a more anterior vulval position (V = 26–29 vs. 48%). Compared to *P. esci*, *P. cantabronavarrus* sp. nov. exhibits a longer odontostyle (158–169 vs. 145–150 μm), a more anterior vulval position (V = 26–29 vs. 48–52%), and longer spicules (94–102 vs. 88–93 µm). It differs from *P. sacchari* by a longer odontostyle (158–169 vs. 105–114 μm), a more anterior vulval position (V = 26–29 vs. 47–52%), a lower c’ ratio (0.5–0.7 vs. 1.0), and the presence of males (vs. absence). Finally, *P. cantabronavarrus* sp. nov. can be distinguished from *P. sali* by its longer body (4.8–5.7 vs. 2.3–2.9 mm), longer odontostyle (158–169 vs. 98–107 μm), and a more anterior vulval position (V = 26–29 vs. 50–54%).

According to the polytomous key by Escuer and Arias [18], the diagnostic codes for *P. cantabronavarrus* sp. nov. are: A1–B1–C1–D23–E2–F2–G7–H1–I3–J1–K3–L1–M3–N34–O2 (with codes in parentheses indicating exceptions).

#### Description

Female. Body moderately long and robust (4.8–5.7) mm, tapering slightly anteriorly. When heat-relaxed, it appears nearly straight or slightly curved ventrally. The cuticle is smooth and relatively thick, with fine transverse striations; thickness 4.5–5.0 µm at mid-body and 13–17 µm at the tail tip. Lip region conoid-rounded and continuous with the rest of the body. Amphidial fovea large and stirrup-shaped, with a conspicuous amphidial aperture measuring 13–14 µm in width, approximately 0.7–0.8 times the diameter of the lip region. Guiding ring located 2.0–2.1 times lip region diameter from anterior end (Table 2). Odontostyle straight or slightly curved distally, relatively long and narrow, measuring 2.9–3.3 times the length of the odontophore. Odontophore moderately developed, with slight swelling of basal musculature (Fig. 3E). Nerve ring encircles the anterior narrower section of pharynx at the mid-point. Pharynx dorylaimoid, typical of genus, with a slender anterior portion that usually loops and overlaps the basal bulb. Basal bulb cylindrical, measuring 126–148 µm in length and 38–52 µm in width. The dorsal pharyngeal gland nucleus (DN) and the ventro-sublateral pair of nuclei (SN) are located at 17.8–23.6%, and 49.8–50.7% of the distance from the anterior end of the pharyngeal bulb, respectively. Glandularium 108–129 µm long. Cardia conoid-rounded, 25–26 µm long. Intestine uniform throughout its length, with a prerectum of variable size. Rectum approximately equal in length to the tail. Reproductive system didelphic-amphidelphic, with two genital branches; the anterior branch is functional but less developed than the posterior one, measuring 420–484 µm, 723–823 µm long, respectively (Table 2). Anterior ovary directed forward, while the posterior ovary is reflexed. Both contain oocytes arranged in a single row, measuring 54–62 µm, 53–61 µm in length, respectively. Uteri measure 76–78 μm and 134–136 μm in length, respectively; sperm cells were observed in several females examined. Vulva appears as a transverse slit, located anterior to the first third of the body. Vagina perpendicular to the body axis, 24–25 μm long, surrounded by well-developed musculature. Tail convex-conoid with a broadly rounded terminus, bearing two or three pairs of caudal pores.

Male. Rare in both populations, with a single specimen detected at each locality. General morphology like that of females, except for genital system. The body is nearly straight, though more curved ventrally in the posterior region. Testes diorchic and opposed, typical of the genus. Spicules paired, dorylaimoid, accompanied by well-developed protractor and retractor muscles. Lateral guiding pieces ventrally curved, measuring 19–21 μm. Cloacal supplements paired, preceded anteriorly by a row of 17 irregularly spaced ventromedians supplements. Tail dorsally convex with broadly rounded terminus. Tail length slightly less than cloacal body width.

Juveniles. Morphologically resemble adults except for body size, tail shape, an undeveloped reproductive system, and the presence of a replacement odontostyle. The four-juvenile life-stages (J1–J4) were distinguishable based on the relative lengths of the functional and replacement odontostyles, as well as overall body length. In J1, the tip of the replacement odontostyle is located near the base of the functional odontostyle. In contrast, in J2–J4, the replacement odontostyle is positioned considerably farther from the functional odontostyle. A scatter diagram illustrating the relationships among functional odontostyle length, replacement odontostyle length, and body length in both juveniles and females is presented in Fig. 4. The tail of the J1 is short-cylindrical with a broadly rounded terminus, and is approximately equal in length and width at the level of the anus. In contrast, the tail of J2 to J4 is rounded and showe a c’ value below 1.0 (Figs. 2, 3).

### Molecular characterization of *Paralongidorus cantabronavarrus* sp. nov

Amplification of the D2–D3 expansion segments of the 28S rRNA, ITS rRNA, partial 18S rRNA, and the mitochondrial *COI* gene from both populations of *P. cantabronavarrus* sp. nov. yielded single fragments of approximately 800 bp, 1100 bp, 1800 bp, and 400 bp long, respectively, as estimated by gel electrophoresis. In total, 12 sequences of the 28S D2–D3 expansion segments were obtained (six from Soba, Cantabria, and six from Elizondo, Navarra; accession numbers PX369417–PX369428). Additionally, seven ITS rRNA sequences (four from Soba, Cantabria, and three from Elizondo, Navarra; accession numbers PX369429–PX369435), three partial 18S rRNA sequences (two from Soba, Cantabria, and one from Elizondo, Navarra; accession numbers PX369436–PX369438), and eight *COI* sequences (six from Soba, Cantabria, and two from Elizondo, Navarra; accession numbers PX369900–PX369907) were generated (Table 1).

Very low intraspecific sequence variation was observed in the D2–D3 expansion segments of *P. cantabronavarrus* sp. nov. (PX369417–PX369428), with differences of only 0–1 bp and 0–1 indels, corresponding to 99.9% sequence identity. In comparison with other *Paralongidorus* species available in GenBank, the D2–D3 sequences of *P. cantabronavarrus* sp. nov. (PX369417–PX369428) exhibited the following levels of identity: 96.3–96.4% to D2–D3 sequences of *P. maximus* (AF480083, KF412826), differing by 27–28 bp and 0–1 indels (Poland [106]); 96.1% to D2–D3 sequences of *Paralongidorus* sp. YH2004 (AY601582), differing by 29 bp and 1 indel (Slovakia [36]); 95.6–95.8% to D2–D3 sequences of *P. rex* (KJ427791–KJ427793), differing by 29–31 bp and 1–3 indels (Poland and Ukraine [107]); 94.8% to D2–D3 sequences of *P. francolambertii* (LT669805), differing by 39 bp and 7 indels (Serbia [22]); 94.4–94.7% to D2–D3 sequences of *P. plesioepimikis* (JQ673403, KY750563–KY750564), differing by 40–42 bp and 1–5 indels (southern Spain [4]); 94.6% to D2–D3 sequences of *P. paramaximus* (EU026156), differing by 40 bp and no indels (southern Spain); 94.2–94.3% to D2–D3 sequences of *P. lusitanicus* (KY750560–KY750562), differing by 43 bp and no indels (Portugal [23]); 94.2% to D2–D3 sequences of *P. litoralis* (EU026155), differing by 43 bp and 4 indels (southern Spain); 93.6% to D2–D3 sequences of *P. iranicus* (JN032587, PP442018), differing by 48 bp and 4 indels (Iran [21]). These sequences were markedly divergent from those of *Longidorus* species available in NCBI, displaying less than 88% identity, with differences exceeding 95 bp and more than 13 indels.

The ITS rRNA region was successfully amplified from seven specimens of *P. cantabronavarrus* sp. nov. (PX369429–PX369435), exhibiting low intraspecific variation, with differences ranging from 3 to 24 bp and 0–2 indels (99.9–100% identity). Comparative analysis with ITS sequences of other *Paralongidorus* species available in GenBank revealed the following levels of identity: 80.4% to those of *P. iranicus* (JN032588), differing by 160 bp and 73 indels (Iran [21]); 79.0% to those of *P. francolambertii* (LT669804), differing by 295 bp and 111 indels (Serbia [22]); 78.6% to those of *P. paramaximus* (JQ673410), differing by 303 bp and 127 indels (Spain [20]); and 77.2–77.3% to those of *P. rex* (KM103254–KM103257), differing by 251–252 bp and 98–100 indels (Poland and Ukraine [107]). All other available ITS sequences of *Paralongidorus* and *Longidorus* species showed less than 45% coverage when aligned with newly generated sequences of *P. cantabronavarrus* sp. nov., indicating substantial genetic divergence.

Three partial 18S rRNA sequences from *P. cantabronavarrus* sp. nov. (PX369436–PX369438) displayed no intraspecific variation (100% identity), regardless of locality. Comparative analysis with sequences available in GenBank revealed high level of identity (98–99%) with several sequences of *Paralongidorus* species: 99.4% identity to those of *P. maximus* (AJ875152), differing by 10 bp and one indel (Germany [108]), 99.2% to those of *P. litoralis* (EU026158), differing by 13 bp and one indel (Spain [20]), 99.2% to those of *P. iranicus* (JN032589), differing by 14 bp and four indels (Iran [21]), 98.9% to those of *P. paramaximus* (EU026157), differing by 17 bp and two indels (Spain [20]), 98.6–98.9% to those of *P. sali* (MG729696–MG729697), differing by 19–24 bp and 3–7 indels (China [109]). Slightly lower identity was observed with sequences of *Longidorus* species, including 98.3–98.4% to those of *Longidorus grandis* Ye and Robbins, 2003 [110] and *Longidorus ferrisi* Robbins, Ye and Pedram, 2009 [111] (AY283165, AY283163), differing in 27–30 bp and two indels (Arkansas and California, USA [111, 112]).

The mitochondrial *COI* region of *P. cantabronavarrus* sp. nov. (PX369900–PX369907) showed low identity with those of other *Paralongidorus* species available in GenBank ranging from 74.6% to 77.8%, with sequence differences of 75–86 bp and no indels. These comparisons included *P. litoralis*, *P. iranicus*, *P. bikanerensis* and *P. paramaximus* from Spain and Iran [21, 113]. Comparable levels of divergence were observed with species from the genera *Longidorus* Micoletzky, 1922, and *Xiphinema* Cobb, 1913 showing differences of 61–87 bp and 0–8 indels, with overall sequence identity ranging from 74.8 to 80.6%. These comparisons included *L. cretensis* Tzortzakakis, Peneva, Terzakis, Neilson, Brown, 2001 [114] *, L. vineacola* Sturhan and Weischer, 1964 [115]*, L. jonesi* Siddiqi, 1962 [116], *L. iranicus* Sturhan and Barooti, 1983 [117], *X. celtiense* Archidona-Yuste, Navas-Cortés, Cantalapiedra-Navarrete, Palomares-Rius & Castillo, 2016 [34]*, X. setariae* Tarjan, 1964 [118], *X. lambertii* Bajaj and Jairajpuri, 1976 [119], from Costa Rica, Czech Republic, Greece, Japan, Spain and Iran [120–122].

### Phylogenetic relationships among *Paralongidorus* and *Longidorus* species

**Fig. 5:**
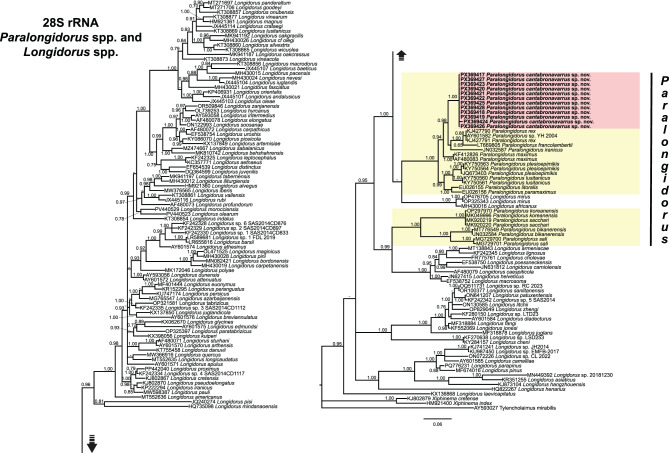
Phylogenetic relationships of *Paralongidorus cantabronavarrus* sp. nov.with *Longidorus* spp. and *Paralongidorus* spp. from NCBI. Bayesian 50% majority rule consensus tree as inferred from D2–D3 expansion segments of 28S rRNA gene sequence alignment under the symmetrical model with invariable sites and gamma distribution (SYM + I + G). Posterior probabilities of more than 0.70 are given for appropriate clades. Newly obtained sequences in this are shown in bold. The scale bar indicates expected changes per site, and the coloured boxes indicate the clade association within *Paralongidorus* species analysed in this study

**Fig. 6:**
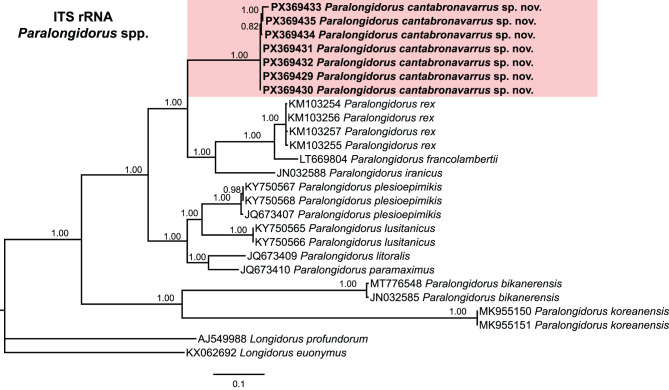
Phylogenetic relationships among *Paralongidorus cantabronavarrus* sp. nov. and *Paralongidorus* spp. from NCBI. Bayesian 50% majority rule consensus tree as inferred from ITS rRNA gene sequence alignment under the General time-reversible model with a gamma distribution (GTR + G). Posterior probabilities of more than 0.70 are given for appropriate clades. Newly obtained sequences in this are shown in bold. The scale bar indicates expected changes per site, and the coloured boxes indicate the clade association within *Paralongidorus* species analysed in this study

**Fig. 7:**
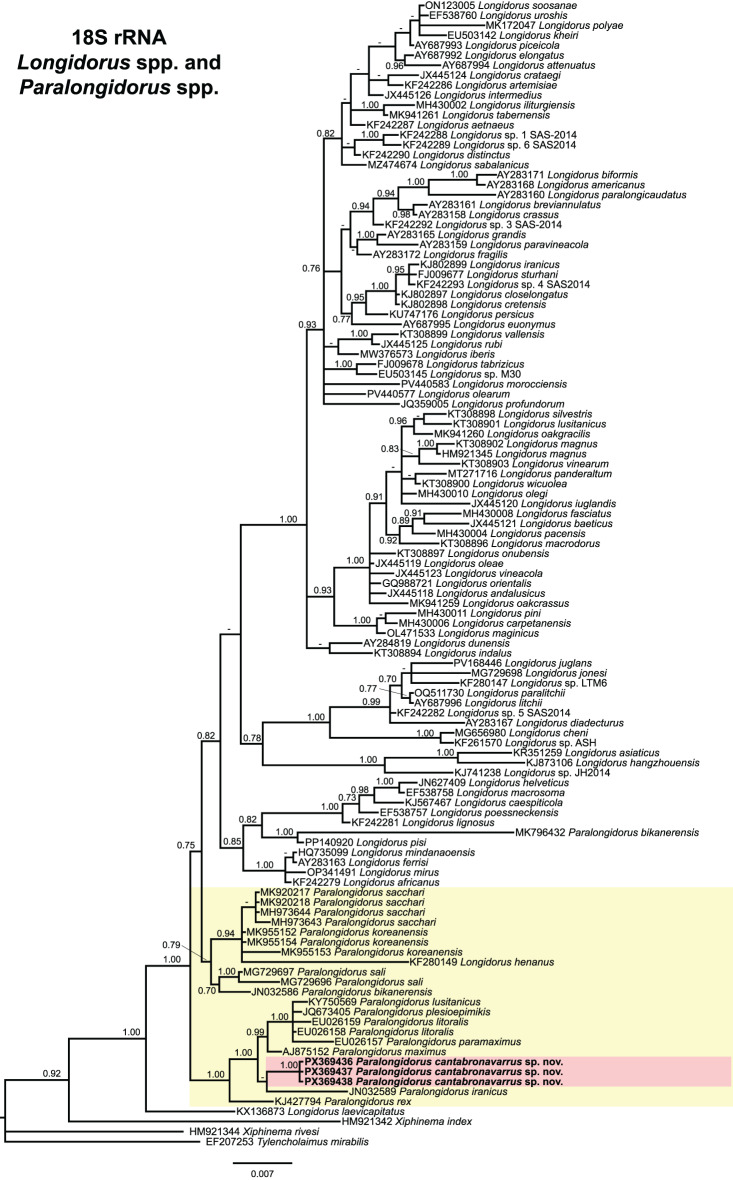
Phylogenetic relationships of *Paralongidorus cantabronavarrus* sp. nov. with *Longidorus* spp. and *Paralongidorus* spp. from NCBI. Bayesian 50% majority rule consensus tree as inferred from partial 18S rRNA gene sequence alignment under the General time-reversible model with a gamma distribution (GTR + G). Posterior probabilities of more than 0.70 are given for appropriate clades. Newly obtained sequences in this are shown in bold. The scale bar indicates expected changes per site, and the coloured boxes indicate the clade association within *Paralongidorus* species analysed in this study

**Fig. 8:**
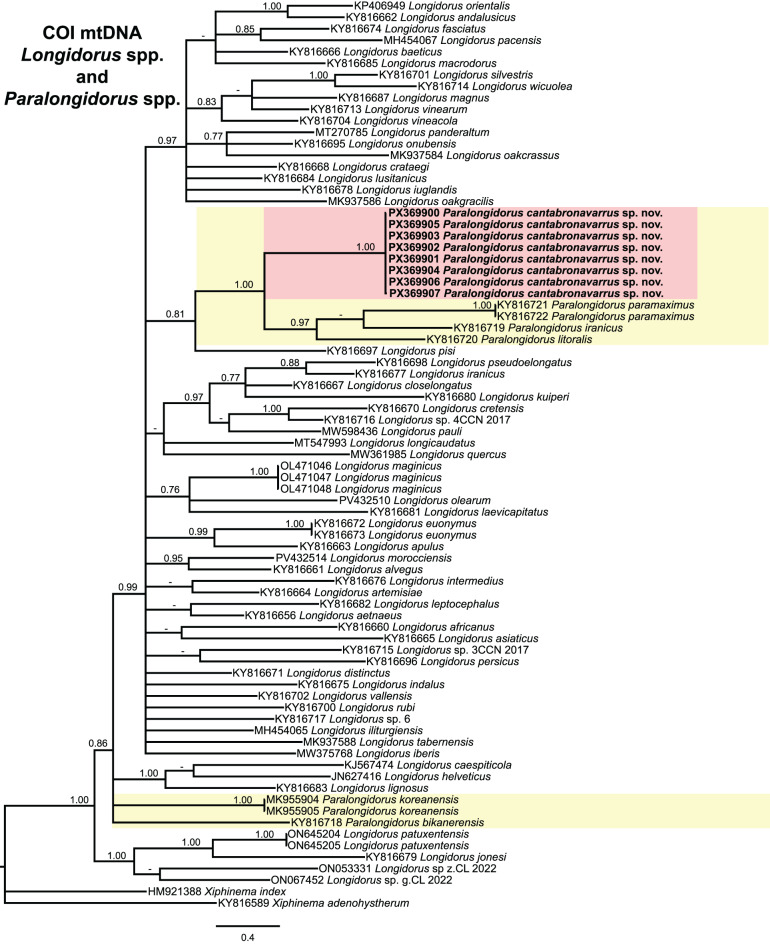
Phylogenetic relationships of *Paralongidorus cantabronavarrus* sp. nov. with *Longidorus* spp. and *Paralongidorus* spp. from NCBI. Bayesian 50% majority rule consensus tree as inferred from *COI* mtDNA gene sequence alignment under General time-reversible model with invariable sites and gamma distribution (GTR + I + G). Posterior probabilities of more than 0.70 are given for appropriate clades. Newly obtained sequences in this are shown in bold. The scale bar indicates expected changes per site, and the coloured boxes indicate the clade association within *Longidorus* species analysed in this study

The phylogenetic relationships of newly identified populations of needle nematodes belonging to *Paralongidorus* were inferred using BI based using sequences of the D2–D3 expansion segments of the 28S rRNA, ITS rRNA, partial 18S rRNA, and mitochondrial *COI* genes (Figs. 5–8).

The D2–D3 alignment (751 bp) comprised 122 *Longidorus* and 34 *Paralongidorus* sequences, along with three sequences of outgroup taxa: *Tylencholaimus mirabilis* (Bütschli, 1873) De Man, 1876 [123, 124] (AY593027), *Xiphinema index* Thorne and Allen, 1950 [125] (HM921400), and *Xiphinema cretense* Tzortzakakis et al. 2014 [126] (KJ136868). Twelve novel sequences generated in this study were included (Fig. 5). The resulting 50% majority-rule consensus tree placed the 34 *Paralongidorus* sequences into two distinct subclades within the principal Clade I of *Longidorus* spp. *sensu* Subbotin et al. [127]. The newly obtained accessions of *P. cantabronavarrus* sp. nov. (PX369417–PX369428) formed a well-supported subclade (PP = 1.00), separated from previously described *Paralongidorus* species, including *P. rex*, *P. francolambertii*, *P. iranicus*, *P. maximus*, *P. plesioepimikis*, *P. lusitanicus*, *P. litoralis*, and *P. paramaximus* (Fig. 5). This subclade was in poorly supported association with a subclade comprising sequences of *L. africanus* Merny, 1966 [128] and *L. mirus* Khan, Chawla and Seshadri, 1972 [129] (Fig. 5). In contrast, four other *Paralongidorus* species (*P. koreanensis*, *P. sacchari*, *P. bikanerensis*, and *P. sali*) clustered separately within a well-supported subclade, which was associated with a well-supported subclade (PP = 0.99) containing eight *Longidorus* species: *L. armeniacae* Bakhshi Amrei, Peneva, Rakhshandehroo, Pedram. 2022 [130], *L. lignosus* Chizhov, Subbotin, Romanenko, Kruchina, 1991 [131], *L. cholevae* Peneva, Lazarova, De Luca and Brown, 2013 [132], *L. poessneckensis* Altherr, 1974 [133], *L. carniolensis* Sirca, Urek, Lazarova, Elshishka and Peneva, 2011 [134], *L. caespiticola* Hooper, 1961 [135], *L. helveticus* Lamberti, Kunz, Grunder, Molinari, De Luca, Agostinelli and Radicci, 2001 [136], and *L. macrosoma* Hooper, 1961 [135] (Fig. 5).

Due to the high variability observed in the ITS sequences between *P. cantabronavarrus* sp. nov. (PX369429–PX369435) and species of *Longidorus*, the ITS rRNA gene phylogeny of *P. cantabronavarrus* sp. nov. was inferred using only *Paralongidorus* sequences available in GenBank. The ITS rRNA gene alignment, comprising 1735 bp, included 24 ingroup sequences and two outgroup sequences belonging to *Longidorus profundorum* Hooper, 1965 [137] and *L. euonymus* Mali and Hooper, 1974 [138] (AJ549988, KX062692). The Bayesian 50% majority-rule consensus tree derived from this alignment is presented in Fig. 6. Bayesian inference of this marker resolved *P. cantabronavarrus* sp. nov. within a well-supported clade (PP = 1.00), distinct from all other *Paralongidorus* species with available sequences. This clade also included *P. iranicus*, *P. francolambertii*, and *P. rex* (Fig. 6).

The partial 18S rRNA gene alignment comprised 112 sequences, spanning 1714 bp, and included sequences of three outgroups: *Tylencholaimus mirabilis* (EF207253), *Xiphinema rivesi* Dalmasso, 1969 [75] (HM921344), and *Xiphinema index* (HM921342) (Fig. 7). *Paralongidorus cantabronavarrus* sp. nov. (PX369436–PX369438) formed a well-supported subclade, distinct from other *Paralongidorus* species, and was nested within a robust clade comprising *P. lusitanicus*, *P. plesioepimikis*, *P. litoralis*, *P. paramaximus*, *P. maximus*, *P. rex*, and *P. iranicus*, although the phylogenetic position of the latter was poorly supported (Fig. 7). A separate, poorly resolved clade included additional *Paralongidorus* species, such as *P. sacchari*, *P. koreanensis*, *P. sali*, and *P. bikanerensis* and *L. henanus* Xu and Cheng, 1982 [139] (KF280149) (Fig. 7).

Phylogenetic analysis of the mitochondrial *COI* gene was conducted on 77 sequences and a 377 bp alignment, incorporating sequences of two outgroup species: *Xiphinema adenohystherum* Lamberti, Castillo, Gómez-Barcina, Agostinelli, 1992 [140] (KY816589) and *Xiphinema index* (HM921388) (Fig. 8). Although the phylogenetic resolution of *COI* was limited, sequences of *P. cantabronavarrus* sp. nov. (PX369900–PX369907) formed a well-supported subclade and were nested within a robust clade comprising *P. paramaximus*, *P. iranicus*, and *P. litoralis*. *Paralongidorus koreanensis* and *P. bikanerensis* occupied separate positions from this major clade with *Paralongidorus* species (Fig. 8).

## Discussion

This study primarily aimed to revise the taxonomic status of *Paralongidorus*, providing an updated list of valid nominal species that incorporates recent descriptions and reassignments of certain species to *Longidorus*. A further objective was to update the polytomous key of Escuer and Arias [18], thereby facilitating species identifications using classical tools. Additionally, the research clarified the biodiversity and molecular phylogeny of *Paralongidorus* populations from forested areas of northern Spain, identifying two distinct populations and underscoring the value of integrative taxonomy in disentangling the species-level diversity within this morphologically conservative genus. As a result, *P. cantabronavarrus* sp. nov. is described.

The discovery of *P. cantabronavarrus* sp. nov. contributes to the expanding diversity of the genus *Paralongidorus*, which now comprises 76 described species worldwide. The new species is distinguished by a distinctive combination of morphological traits, notably the anterior vulval position, odontostyle length, amphidial fovea morphology, and guiding ring position, which do not overlap with any previously described species. Recovered from forested regions in northern Spain, its presence extends the known European distribution of the genus, historically underrepresented in this area. The prevailing biogeographical pattern of *Paralongidorus*, with most species occurring in Asia and Africa, supports the hypothesis proposed by Coomans [14], suggesting that the ancestral centre of origin for *Paralongidorus* lies within the region extending from South-East Africa to India. This view suggests the genus likely evolved prior to the fragmentation of Pangaea [141].

Recent studies on Longidoridae, including the present work, indicate that species diversity within *Paralongidorus* remains underestimated due to cryptic taxa [34, 38, 142]. High diversity detected in certain areas, such as India, South Africa, and the Iberian Peninsula suggests potential centres of diversification. To test this hypothesis, the first essential step would be to determine the existing biodiversity of the genus *Paralongidorus* in these areas. This requires intensive nematological surveys supported by an integrative taxonomic approach that combines morphological and molecular analyses [34, 38, 142].

Although *P. cantabronavarrus* sp. nov. was not directly linked to damage in common beech, members of *Paralongidorus* are known ectoparasites and potential nepovirus vectors. This represents the second global record of a *Paralongidorus* species associated with common beech, following *P. maximus* in northern Portugal [143], though that record requires confirmation via integrative taxonomy. Further ecological studies are needed to evaluate the role of *P. cantabronavarrus* sp. nov. in soil ecology and its interactions with common beech. Its detection in natural forest ecosystems raises questions about host specificity, feeding behaviour, and potential impacts on native and cultivated flora. The newly described species exhibits a moderately sized body and an elongated odontostyle, features that may reflect adaptations to feeding on woody hosts such as common beech, consistent with previous hypotheses linking stylet length to host tissue characteristics [34]. However, further nematological surveys across the Iberian Peninsula are essential to clarify its role and ecological niche, as well as to determine whether *P. cantabronavarrus* sp. nov. is endemic of this region.

Taxonomic ambiguities within *Paralongidorus* and its junior synonyms may be clarified through molecular analyses employing ribosomal and mitochondrial markers. Early phylogenetic studies by He et al. [36], based on the D2–D3 expansion segments of 28S rDNA, supported the monophyly of *Paralongidorus*, although only two species were included at that time. Subsequent investigations encompassing a broader representation of *Paralongidorus* species have demonstrated that is not the case [4, 20–24]. Moreover, none of these studies support the validity of *Longidoroides*. Monophyly of *Paralongidorus* was previously hypothesised by Coomans [16] based on morphological characters, and later supported by He et al. [36] using the ribosomal marker (D2–D3). However, the phylogenetic analyses conducted in the present study, based on ribosomal (D2–D3 expansion segments of 28S, ITS and partial 18S) and mitochondrial (partial *COI*) markers with including sequences of *Longidorus* and *Paralongidorus* (Figs. 5–8), do not support the monophyly of *Paralongidorus*. All markers consistently reveal two distinct subclades comprising species currently assigned to the genus. Our analyses consistently placed *P. cantabronavarrus* sp. nov. within a well-supported subclade, although its position in the *COI*-based tree was less resolved and poorly supported compared to ribosomal markers. This genus placement is congruent with previous phylogenetic studies for this molecular marker [4, 20–24].

*Paralongidorus cantabronavarrus* sp. nov. is clearly differentiated from all other congeners and clusters within a subclade comprising species with stirrup-shaped amphidial fovea, including *P. francolambertii, P. iranicus, P. litoralis, P. lusitanicus, P. maximus, P. paramaximus, P. plesioepimikis,* and *P. rex* (Figs. 5–8). In contrast, the second subclade includes species with funnel-shaped amphidial fovea (*P. bikanerensis* and *P. sali*) and stirrup-shaped amphidial fovea (*P. koreanensis* and *P. sacchari*) (Figs. 5–8). These findings support the hypothesis proposed by He et al. [36], suggesting that amphidial fovea morphology may have evolved multiple times within longidorid nematodes. Furthermore, our data reinforce the distinction between *Paralongidorus* and *Longidorus*, in agreement with Palomares-Rius et al. [20], and contradict the proposal by Decraemer and Coomans [6] and Kornobis et al. [107], who considered *P. bikanerensis* a member of *Longidoroides*.

Notably, this is the first report of a *Paralongidorus* species exhibiting the most anterior vulval position (26–29%), implying a reduced but functional anterior genital branch. All other species in the genus showed an almost equatorial vulval position, with few exceptions showing slightly more anterior placements, such as *P. plesioepimikis* (33–38%) [4], *P. namibiensis* (33–44%) [71], *P. maximus* (36–40%) [144]. An anterior vulval position is considered a derived character and may reflect unique developmental or ecological adaptations. It may result from accelerated evolutionary processes or mutations affecting regulatory genes as studied in model taxa (e.g. *lin-11* in *Pristionchus pacificus* Sommer, Carta, Kim & Sternberg, 1996 [145, 146]), which influence cell migration along the vulval precursor axis. Such positioning could affect mating behaviour or facilitate oviposition in microhabitats with spatial constraints, particularly in soil environments with complex root architecture. In ectoparasitic nematodes, anterior vulval placement may reduce interference between reproductive and feeding structures, enhancing host interaction efficiency [147–149].

## Conclusion

This study provides robust molecular and morphological evidence that *Paralongidorus* is paraphyletic, with species consistently resolving into two distinct subclades. The newly described *P. cantabronavarrus* sp. nov. contributes to a more comprehensive understanding of amphidial fovea variation and its phylogenetic significance. The unprecedented anterior vulval position observed in this species may represent a derived developmental trait, potentially linked to ecological adaptation or regulatory gene divergence. These findings reinforce the distinction between *Paralongidorus* and *Longidorus*., and underscore the importance of integrative approaches in resolving long-standing taxonomic ambiguities within Longidoridae. Further genomic and developmental studies will be essential to elucidate the evolutionary mechanisms underlying morphological diversity in this group.

## Electronic supplementary material

Below is the link to the electronic supplementary material.

Supplementary material 1

Supplementary Material 2

## Data Availability

All data generated or analysed during this study are included in this published article. Sequences are deposited in GenBank, NCBI. This article has been registered at Zoobank (http://zoobank.org/urn:lsid:zoobank.org:pub: 04EC6BC9-ED7D-4185-87F8-A8849F91B3B0).
