# Supplementary material for: Systematic revision of Paralongidorus (Nematoda: Longidoridae) based on molecular and morphological evidence, with the description of a new species from Spain
Source: Zoological Lett. 2026 Feb 7;12:6. doi: 10.1186/s40851-026-00259-6 (PMC13041179; doi:10.1186/s40851-026-00259-6)
Supplement: Supplementary file 1 — Supplementary material 1 [file 40851_2026_259_MOESM1_ESM.docx]

**Table S1.** Global distribution of *Paralongidorus* species by continent (number of species in parentheses), listed in alphabetical order*.*

| **Africa**  **(32)** | **North America**  **(1)** | **South and Central America (1)** | **Asia**  **(37)** | | **Oceania**  **(4)** | **Europe**  **(10)** | |
| --- | --- | --- | --- | --- | --- | --- | --- |
| *bikanerensis, bullatus, capensis, cebensis, christiani, clavicaudatus, costatus, dakarensis, deborae, duncani, epimikis, erriae, fischeri, hanliae, hooperi, latilabiatus, lutosus, maximus, namibiensis, natalensis, paramaximus, pulcher, pulcheroides, sandellus, silvestris, spasskii, spaulli, strelitziae, utriculoides, wiesae, xiphinemoides, Paralongidorus* sp. | *microlaimus* | *sacchari* | *afzali, agni, beryllus, bikanerensis, buchae, buckeri, ciaressi, citri, dasturi, distinctus, esci, eugeni, fici, flexus, gloriosus, inagreinus, indicus, koreanensis, lemoni, longiurus, lutensis, major, maximus, mediensis, microlaimus, nudus, oryzae, rex, rotundatus, sacchari, sali, sativus, seclipsi, similis, teres, zenobiae,* *Paralongidorus* sp. | | *australis,*  *eucalypti,*  *sacchari*  *Paralongidorus sp.* | ***cantabronavarrus*** sp. nov., *francolambertii, georgiensis, litoralis, lusitanicus, nudus, paramaximus, plesioepimikis, remyi, rex* | |
| **References** |  |  | |  |  | |  |
| [Corbett, 1964, Heyns, 1965, 1966, 1972, Reis, 1982, Dalmasso, 1969, Jacobs & Heyns, 1982, 1987, Sharma & Siddiqi, 1990, Liebenberg et al. 1993a,b, Siddiqi et al. 1993, Swart et al. 1996, Marais et al., 2004, Faye & Mounport, 2007, 2010, Fourie et al. 2017] | [Robbins, 1978] | [Roca & Rios, 2006] | | [Siddiqi et al., 1963, Siddiqi, 1964, Khan, 1964, Siddiqi & Husain, 1965, Khan et al., 1972, 1976, Chawla & Samathanam, 1981, Khan, 1982, Khan et al., 1980, Baqri & Jairajpuri, 1981, Phukan & Sanwal, 1983, Sharma & Edward, 1985, Khan, 1986, Lamberti et al, 1985, 1999, Brown et al., 1990, Hunt & Rahman, 1991, Nasira et al., 1993, Pedram et al., 2012, Bohra, 2012, Cai et al., 2018, Mwamula et al. 2020] | [Fisher, 1964, Reay, 1987, Stirling and McCulloh, 1984] | | [Tulaganov, 1937, Sturhan, 1963, Heyns, 1975, Andrassy, 1986, Brown et al., 1990, Liskova & Brown, 2003, Barsi et al., 2007, Palomares-Rius et al., 2008, 2013, Tzortzakakis et al., 2008, Peneva et al., 2012, Kornobis et al., 2015, Barsi & De Luca, 2017, Gutiérrez-Gutiérrez et al., 2018, Rubtsova et al., 2001, Susulowska, 2020] |
