# Supplementary material for: Systematic revision of Paralongidorus (Nematoda: Longidoridae) based on molecular and morphological evidence, with the description of a new species from Spain
Source: Zoological Lett. 2026 Feb 7;12:6. doi: 10.1186/s40851-026-00259-6 (PMC13041179; doi:10.1186/s40851-026-00259-6)
Supplement: Supplementary file 2 — Supplementary Material 2 [file 40851_2026_259_MOESM2_ESM.docx]

**Table S2**. Updated polytomous key for *Paralongidorus* Siddiqi, Hooper and Khan, 1963, revised from Escuer and Arias [1997], incorporating newly described species and excluding those reassigned to *Longidorus*. This key is intended to facilitate contemporary morphological and morphometric identifications.

| **Nº** | **SPECIES** | **A** | **B** | **C** | **D** | **E** | **F** | **G** | **H** | **I** | **J** | **K** | **L** | **M** | **N** | **O** |
| --- | --- | --- | --- | --- | --- | --- | --- | --- | --- | --- | --- | --- | --- | --- | --- | --- |
| **1** | *afzali* | **3** | **1** | **1** | **1** | **1** | **12** | **4** | **1** | **4** | **4** | **2** | **23** | **1** |  |  |
| **2** | *agni* | **1** | **1** | **1** | **2** | **2** | **2** | **12** | **12** | **1** | **1** | **1** | **1** | **13** | **1** | **1** |
| **3** | *australis* | **1** | **1** | **1** | **3** | **2** | **56** | **67** | **3** | **12** | **1** | **34** | **13** | **23** | **4** | **3** |
| **4** | *beryllus* | **1** | **1** | **3** | **1** | **1** | **2** | **2** | **1** | **2** | **3** | **3** | **2** | **1** |  |  |
| **5** | *bikanerensis* | **3** | **1** | **3** | **2** | **1** | **34** | **5** | **1** | **2** | **2** | **3** | **3** | **1** |  |  |
| **6** | *buchae* | **1** | **1** | **3** | **2** | **1** | **23** | **4** | **1** | **2** | **2** | **3** | **3** | **2** |  |  |
| **7** | *buckeri* | **1** | **2** | **1** | **2** | **1** | **1** | **1** | **1** | **2** | **2** | **1** | **1** | **1** |  |  |
| **8** | *bullatus* | **1** | **1** | **2** | **1** | **1** | **2** | **57** | **1** | **23** | **3** | **34** | **3** | **12** |  |  |
| **9** | ***cantabronavarrus* sp. nov.** | **1** | **1** | **1** | **23** | **2** | **2** | **7** | **1** | **3** | **1** | **3** | **1** | **3** | **34** | **2** |
| **10** | *capensis* | **13** | **1** | **3** | **2** | **2** | **26** | **45** | **2** | **234** | **3** | **3** | **34** | **123** | **12** | **2** |
| **11** | *cebensis* | **1** | **1** | **1** | **2** | **2** | **4** | **5** | **3** | **2** | **1** | **3** | **1** | **3** | **3** | **3** |
| **12** | *christiani* | **1** | **1** | **3** | **2** | **2** | **3** | **2** | **2** | **2** | **1** | **2** | **1** | **1** | **2** | **2** |
| **13** | *ciaressi* | **1** | **1** | **3** | **2** | **1** | **456** | **34** | **1** | **2** | **23** | **2** | **1** | **2** |  |  |
| **14** | *citri* | **1** | **1** | **3** | **2** | **1** | **4** | **5** | **1** | **2** | **23** | **3** | **3** | **2** |  |  |
| **15** | *clavicaudatus* | **13** | **1** | **1** | **3** | **2** | **2** | **2** | **2** | **12** | **12** | **1** | **12** | **1** | **1** | **1** |
| **16** | *costatus* | **3** | **1** | **1** | **2** | **1** | **2** | **34** | **1** | **24** | **3** | **2** | **23** | **12** |  |  |
| **17** | *dakarensis* | **1** | **1** | **3** | **2** | **1** | **12** | **21** | **1** | **32** | **3** | **12** | **1** | **1** |  |  |
| **18** | *dasturi* | **1** | **2** | **3** | **2** | **1** | **23** | **35** | **1** | **1** | **1** | **3** | **12** | **23** |  |  |
| **19** | *deborae* | **1** | **1** | **3** | **2** | **2** | **6** | **7** | **12** | **2** | **2** | **45** | **4** | **23** | **2** | **23** |
| **20** | *distinctus* | **1** | **1** | **1** | **2** | **1** | **4** | **7** | **2** | **2** | **1** | **4** | **1** | **3** |  |  |
| **21** | *duncani* | **2** | **1** | **2** | **1** | **2** | **1** | **1** | **1** | **34** | **4** | **1** | **1** | **1** | **1** | **12** |
| **22** | *epimikis* | **1** | **1** | **34** | **2** | **2** | **6** | **7** | **1** | **2** | **2** | **4** | **4** | **2** | **1** | **2** |
| **23** | *erriae* | **1** | **1** | **3** | **2** | **2** | **12** | **1** | **1** | **12** | **23** | **2** | **13** | **1** | **1** | **2** |
| **24** | *esci* | **1** | **1** | **1** | **3** | **2** | **2** | **6** | **2** | **2** | **1** | **34** | **1** | **1** | **1** | **3** |
| **25** | *eucalypti* | **1** | **1** | **3** | **23** | **1** | **23** | **56** | **2** | **3** | **3** | **3** | **23** | **12** |  |  |
| **26** | *eugeni* | **3** | **1** | **3** | **2** | **1** | **2345** | **46** | **2** | **1** | **2** | **2** | **13** | **2** |  |  |
| **27** | *fici* | **1** | **1** | **3** | **2** | **1** | **34** | **45** | **1** | **2** | **23** | **3** | **3** | **2** |  |  |
| **28** | *fischeri* | **1** | **2** | **3** | **2** | **2** | **2** | **2** | **12** | **23** | **3** | **2** | **12** | **12** | **1** | **1** |
| **29** | *flexus* | **1** | **1** | **1** | **1** | **2** | **2** | **34** | **1** | **4** | **4** | **2** | **13** | **1** | **1** | **1** |
| **30** | *francolambertii* | **1** | **1** | **4** | **2** | **2** | **345(6)** | **56** | **1** | **1(2)** | **12** | **3** | **34** | **2(3)** | **1** | **1** |
| **31** | *georgiensis* | **1** | **1** | **3** | **2** | **1** | **34** | **5** | **1** | **2** | **2** | **3** | **3** | **2** |  |  |
| **32** | *gloriosus* | **3** | **1** | **3** | **2** | **1** | **34** | **45** | **1** | **2** | **3** | **2** | **3** | **2** |  |  |
| **33** | *halepensis* | **2** | **1** | **1** | **3** | **2** | **45** | **34** | **1** | **2** | **2** | **2** | **3** | **3** | **2** | **1** |
| **34** | *hanliae* | **1** | **1** | **3** | **2** | **2** | **23** | **23** | **1** | **12** | **13** | **23** | **13** |  | **1** | **13** |
| **35** | *hooperi* | **2** | **2** | **2** | **2** | **2** | **56** | **7** | **3** | **23** | **12** | **45** | **4** | **2** | **23** | **12** |
| **36** | *inagreius* | **1** | **1** | **3** | **2** | **1** | **2** | **3** | **1** | **12** | **12** | **3** | **23** | **2** |  |  |
| **37** | *indicus* | **1** | **1** | **3** | **2** | **1** | **45** | **3** | **1** | **2** | **2** | **2** | **3** | **13** |  |  |
| **38** | *iranicus* | **1** | **1** | **4** | **2** | **2** | **6** | **7** | **12** | **22** | **1** | **45** | **23** | **3** | **23** | **2** |
| **39** | *koreanensis* | **1** | **1** | **3** | **3** | **2** | **2** | **3** | **1** | **23** | **23** | **23** | **1** | **23** | **2** | **2** |
| **40** | *latilabiatus* | **3** | **1** | **2** | **2** | **1** | **4** | **2** | **3** | **2** | **3** | **3** | **4** | **1** |  |  |
| **41** | *lemoni* | **1** | **1** | **2** | **1** | **1** | **12** | **1** | **1** | **2** | **4** | **1** | **23** | **1** |  |  |
| **42** | *litoralis* | **1** | **1** | **4** | **2** | **2** | **6** | **7** | **1** | **2** | **1** | **5** | **4** | **2** | **2** | **2** |
| **43** | *longiurus* | **3** | **2** | **12** | **1** | **1** | **1** | **2** | **1** | **4** | **4** | **2** | **1** | **1** |  |  |
| **44** | *lusitanicus* | **1** | **1** | **3** | **2** | **2** | **6** | **7** | **23** | **2(3)** | **1(2)** | **56** | **3(4)** | **2(3)** | **2** | **2** |
| **45** | *lutensis* | **1** | **2** | **1** | **3** | **1** | **35** | **57** | **3** | **1** | **1** | **12** | **1** |  |  |  |
| **46** | *lutosus* | **23** | **1** | **23** | **2** | **2** | **4** | **23** | **12** | **2** | **3** | **2** | **34** | **13** | **1** | **1** |
| **47** | *major* | **1** | **1** | **3** | **2** | **1** | **24** | **4** | **1** | **2** | **2** | **23** | **23** | **2** |  |  |
| **48** | *maximus* | **1** | **1** | **3** | **2** | **2** | **56** | **7** | **2** | **2** | **1** | **6** | **13** | **3** | **4** | **2** |
| **49** | *mediensis* | **1** | **1** | **3** | **2** | **1** | **2** | **45** | **1** | **2** | **3** | **2** | **23** | **1** |  |  |
| **50** | *microlaimus* | **1** | **1** | **1(3)** | **2** | **2** | **12** | **2** | **1** | **12** | **23** | **2** | **1** | **1** | **1** | **1** |
| **51** | *namibiensis* | **1** | **1** | **3** | **2** | **1** | **45** | **67** | **1** | **12** | **13** | **4** | **34** | **12** |  |  |
| **52** | *nudus* | **1** | **1** | **3** | **2** | **1** | **2** | **3** | **1** | **3** | **23** | **2** | **1** | **1** |  |  |
| **53** | *oryzae* | **1** | **1** | **1(3)** | **2** | **1** | **1** | **1** | **1** | **2** | **3** | **1** | **1** | **1** |  |  |
| **54** | *paramaximus* | **1** | **1** | **4** | **2** | **2** | **4** | **4567** | **12** | **12** | **12** | **45** | **34** | **13** | **13** | **23** |
| **55** | *pini* | **3** | **1** | **1** | **2** | **2** | **356** | **34** | **23** | **23** | **3** | **3** | **23** | **23** | **2** | **12** |
| **56** | *plesioepimikis* | **1** | **1** | **4** | **2** | **1** | **6** | **7** | **2** | **23** | **1** | **6** | **4** | **3** |  |  |
| **57** | *pulcher* | **3** | **1** | **1** | **2** | **1** | **2** | **2** | **1** | **12** | **12** | **2** | **13** | **13** |  |  |
| **58** | *pulcheroides* | **3** | **1** | **1** | **2** | **1** | **12** | **1** | **1** | **2** | **3** | **12** | **23** | **1** |  |  |
| **59** | *remyi* | **1** | **1** | **3** | **2** | **1** | **2** | **7** | **1** | **2** | **1** | **1** |  |  |  |  |
| **60** | *rex* | **1** | **1** | **4** | **3** | **1** | **6** | **7** | **2** | **3** | **1** | **6** | **23** | **3** |  |  |
| **61** | *rotundatus* | **1** | **2** | **3** | **3** | **1** | **2** | **67** | **2** | **1** | **1** | **3** | **1** | **1** |  |  |
| **62** | *sacchari* | **1** | **1** | **1** | **2** | **1** | **2** | **34** | **1** | **3** | **23** | **23** | **1** | **2** |  |  |
| **63** | *sali* | **1** | **1** | **1** | **3** | **1** | **1** | **3** | **1** | **1** | **1** | **2** | **1** | **1** |  |  |
| **64** | *sandellus* | **2** | **1** | **2** | **2** | **2** | **1** | **2** | **3** | **1** | **23** | **2** | **1** | **1** | **1** | **1** |
| **65** | *sativus* | **2** | **2** | **1** | **2** | **1** | **2** | **1** | **2** |  | **2** | **2** | **1** | **1** |  |  |
| **66** | *seclipsi* | **1** | **1** | **3** | **2** | **2** | **34** | **56** | **1** | **2** | **1** | **4** | **12** | **3** | **2** | **2** |
| **67** | *silvestris* | **3** | **2** | **3** | **1** | **2** | **2** | **2** | **1** | **34** | **34** | **2** | **23** | **1** | **1** | **1** |
| **68** | *similis* | **1** | **1** | **1** | **2** | **1** | **1** | **1** | **1** | **1** | **2** | **1** | **1** | **1** |  |  |
| **69** | *spasskii* | **1** | **1** | **3** | **2** | **2** | **34** | **2** | **1** | **2** | **2** | **34** | **23** | **12** | **12** | **2** |
| **70** | *spaulli* | **1** | **1** | **3** | **3** | **2** | **23** | **12** | **1** | **12** | **3** | **1** | **23** | **1** | **1** | **13** |
| **71** | *strelitziae* | **13** | **2** | **1** | **23** | **2** | **26** | **25** | **3** | **12** | **1** | **34** | **2** | **3** | **23** | **23** |
| **72** | *teres* | **3** | **1** | **3** | **2** | **1** | **24** | **45** | **1** | **2** | **2** | **3** | **13** | **2** |  |  |
| **73** | *utriculoides* | **3** | **1** | **1** | **2** | **1** | **2** | **1** | **1** | **2** | **3** | **2** | **3** | **1** |  |  |
| **74** | *wiesae* | **3** | **1** | **1** | **2** | **1** | **2** | **34** | **2** | **23** | **12** | **23** | **1** | **23** |  |  |
| **75** | *xiphinemoides* | **1** | **1** | **2** | **2** | **1** | **12** | **3** | **3** | **12** | **3** | **2** | **12** | **1** |  |  |
| **76** | *zenobiae* | **1** | **2** | **1** | **3** | **1** | **46** | **7** | **3** | **24** | **1** | **1** | **1** | **1** |  |  |

|  | **Not available** |
| --- | --- |

**(codes in parentheses are exceptions)**

Escuer M, Arias M. *Paralongidorus iberis* sp. n and *P. monegrensis* sp. n from Spain with a polytomous key to the species of the genus *Paralongidorus* Siddiqi, Hooper & Khan, 1963 (Nematoda: Longidoridae). Fundam. Appl. Nematol. 1997; 20:135-148.

Siddiqi MR, Hooper DJ, Khan E. A new nematode genus *Paralongidorus* (Nematoda: Dorylaimoidea) with description of two new species and observations on *Paralongidorus citri* (Siddiqi, 1959) n. comb. Nematologica. 1963; 9:7-14.

Diagnostic character states used in the polytomous key for *Paralongidorus* species identification. Each trait is assigned a set of coded values representing discrete morphological and morphometric features, facilitating comparative analysis and species-level resolution.

| **A=amphidial pouches** |  | **F=body length females** |  | **I= tail length** |  | **L= ratio a** |
| --- | --- | --- | --- | --- | --- | --- |
| 1= funnel or stirrup-shape |  | 1= <3,6 mm |  | 1= < 27 |  | 1= < 95 |
| 2=cup-shaped |  | 2= 3,6-5,6 mm |  | 2= 27-38 |  | 2= 95-108 |
| 3=pouch-shaped |  | 3= 5,7-6,2 mm |  | 3= 39-50 |  | 3= 109-145 |
| **B= width amphidial fovea** |  | 4= 6,3-7,6 mm |  | 4= >50 |  | 4= >145 |
| 1= half or more as wide as lip region |  | 5= 7,7-8,2 mm |  | **J= c'** |  | **M= basal bulb** |
| 2= less than half |  | 6= > 8,2 mm |  | 1= <0,8 |  | 1= < 108 |
| **C=Lipregion shape** |  | **G= odontostyle** |  | 2= 0,8-1,0 |  | 2= 108-128 |
| 1=continuous |  | 1= < 70 |  | 3= 1,1-1,8 |  | 3= >128 |
| 2=expanded |  | 2= 70-94 |  | 4= >1,8 |  | **N= spicules** |
| 3=set off by constriction |  | 3= 95-110 |  | **K=Lip region width** |  | 1= <60 |
| 4= clearly set off by a deep constriction |  | 4= 111-124 |  | 1= < 10 |  | 2= 60-80 |
| **D=Female tail shape** |  | 5= 125-140 |  | 2= 10-15 |  | 3= 81-100 |
| 1=conoid |  | 6= 141-152 |  | 3=16-20 |  | 4= >100 |
| 2=conoid with broad rounded tip |  | 7= > 152 |  | 4= 21-25 |  | **O= supplements** |
| 3=hemispherical |  | **H= Oa-gr** |  | 5= 26-30 |  | 1= <11 |
| **E=Males** |  | 1= <37 |  | 6= >30 |  | 2=11-16 |
| 1= unknown |  | 2= 37-47 |  |  |  | 3= >16 |
| 2= known |  | 3= >47 |  |  |  |  |
